# Integrated genetic and epigenetic analysis identifies that rs939408 affects non-smoking lung adenocarcinoma risk by modulating the DNA methylation of *LRRC2*

**DOI:** 10.1038/s41419-025-08163-1

**Published:** 2025-11-17

**Authors:** Lei Zhang, Zhenyu Li, Yanchi Wang, Mingjiong Zhang, Haoyan Chen, Yifan Cheng, Qiong Chen, Baosheng Cui, Jiahao Liu, Haiyan Gong, Rui Zhu, Tian Tian, Yan Zhang, Shengguang Ding, Yu Duan, Shuangshuang Wu, Minjie Chu

**Affiliations:** 1 Institute for Applied Research in Public Health, Key Laboratory of Jiangsu Higher Education Institutions for Advanced Medical Analytics and Public Health, School of Public Health, Nantong University, Nantong, Jiangsu China; 2 Affiliated Nantong Hospital of Shanghai University (The Sixth People’s Hospital of Nantong), Nantong, Jiangsu China; 3 Jiangsu Provincial Key Laboratory of Geriatrics, Department of Geriatrics, The First Affiliated Hospital of Nanjing Medical University, Nanjing, Jiangsu China; 4 Department of Thoracic Surgery, Affiliated Qidong Hospital of Nantong University, Qidong People’s Hospital, Qidong Liver Cancer Institute, Nantong, Jiangsu China; 5 Department of Thoracic Surgery, The Second Affiliated Hospital of Nantong University, Nantong, Jiangsu China

**Keywords:** Genetics research, Oncogenesis

## Abstract

This study aimed to investigate the relationship between methylation quantitative trait loci (meQTL) and lung adenocarcinoma (LUAD) susceptibility. Candidate SNPs linked to differentially methylated CpG sites in LUAD were identified through meQTL datasets. Genome-wide association study (GWAS) data were analyzed to assess the correlation between selected meQTLs and LUAD risk. The effects of target genes on malignant LUAD phenotypes were examined through both in vitro and in vivo experiments. Additionally, machine learning and radiomics models were employed to evaluate the association of target genes on LUAD progression. The variant A allele of rs939408 was associated with decreased methylation levels of cg09596674 in *LRRC2* (β < 0, *P* < 0.001). While cg09596674 was highly methylated, *LRRC2* showed lower expression in LUAD tumor tissues. Consistently, a negative correlation was observed between methylation of cg09596674 and *LRRC2* expression (r = −0.32, *P* < 0.001), indicating that lower methylation of cg09596674 modulated by rs939408 may reduce non-smoking LUAD risk (OR = 0.89, *P* = 0.019). Increased *LRRC2* expression inhibited LUAD cell line malignancy and suppressed tumor growth in mice. Furthermore, lower *LRRC2* expression was linked to metastasis (*P* = 0.02) and higher levels of two poorer survival-related imaging features (*P* = 0.03). The meQTL rs939408 may modulate DNA methylation of *LRRC2*, thereby influencing its expression and potentially affecting non-smoking LUAD risk. These findings offer valuable insights into the role of meQTLs in LUAD carcinogenesis.

## Introduction

Lung cancer remains the most frequently diagnosed malignancy and the leading cause of cancer-related deaths globally [1]. With rising cases and mortality rates, the burden of lung cancer is increasing in China as well [2]. Non-small cell lung cancer (NSCLC) accounts for 80% to 85% of all lung cancer cases [3], with lung adenocarcinoma (LUAD) and lung squamous carcinoma (LUSC) being the most prevalent histological subtypes [4]. In recent years, LUAD and LUSC have displayed opposing trends in incidence: while LUSC, primarily linked to tobacco use, has been steadily declining, LUAD has shown a notable upward trend [5, 6]. As a result, the prevention and control of LUAD have become an urgent public health priority.

LUAD development is influenced not only by smoking but also by genetic, epigenetic, and other factors [7]. Genetic predisposition plays a pivotal role in the pathogenesis of LUAD [8]. Single nucleotide polymorphisms (SNPs) are significant determinants of genetic susceptibility, with numerous SNPs linked to LUAD risk identified through genome-wide association studies (GWAS) [9, 10]. However, many of these SNPs are located in non-coding regions, and their biological functions in disease mechanisms remain largely unexplored [11, 12].

DNA methylation, a key epigenetic regulatory mechanism, can suppress gene transcription by modifying chromatin structure [13, 14]. Aberrant methylation is closely linked to cancer, often occurring at early stages of tumor development, including in LUAD [15–17]. SNPs may influence gene expression directly or indirectly by altering methylation patterns, thereby affecting lung cancer development [18, 19]. Investigating the relationships and mechanisms among SNPs, DNA methylation, gene expression, and LUAD risk could pave the way for targeted prevention and treatment strategies.

Quantitative trait loci (QTL) analysis has emerged as a powerful approach for assessing the impact of genetic variation on intermediate molecular phenotypes, including expression QTL (eQTL) and methylation QTL (meQTL) analysis [20]. While eQTL analysis has identified certain SNPs associated with LUAD [21], it remains insufficient in elucidating the underlying mechanisms. MeQTL analysis, which assesses the influence of SNPs on methylation at specific loci, is particularly relevant as methylation of promoter regions can regulate gene expression, influencing tumor development. MeQTL analysis thus provides insights into the genetic factors driving individual epigenomic differences [22]. Recently, Gong et al. [23] compiled a meQTL database covering 23 cancer types by integrating genome-wide genotype and DNA methylation data. However, the identified meQTLs are the result of bioinformatics analysis, and their biological relevance has yet to be experimentally validated. As such, their role in disease mechanisms remains uncertain, warranting further exploration before they can be considered reliable biomarkers.

This study aimed to identify and evaluate meQTLs linked to LUAD and investigate their potential pathogenic mechanisms. First, differentially methylated CpG sites associated with LUAD were identified by integrating DNA methylation data from in-house and The Cancer Genome Atlas (TCGA) samples. Next, candidate SNPs correlated with these CpG sites were obtained using meQTL datasets. Subsequently, GWAS data from 3453 non-smoking LUAD cases and 3710 healthy controls were analyzed to assess the relationship between these candidate meQTLs and LUAD risk. Functional assays were then conducted to explore the effects of the target gene corresponding to the meQTL on LUAD malignancy in vitro and in vivo. Lastly, machine learning and radiomics models were employed to evaluate the prognostic significance of the target genes.

## Materials and methods

### Study participants and sample information of in-house and TCGA methylation array

The in-house samples consisted of 10 pairs of matched tumor and adjacent non-tumor tissues from patients with LUAD, collected at the Second Affiliated Hospital of Nantong University. All patients were newly diagnosed based on postoperative pathology and had not received chemotherapy or radiotherapy prior to surgery. External DNA methylation data (455 LUAD tumor tissues and 32 adjacent non-tumor tissues) and gene expression data (510 LUAD tumor tissues and 58 adjacent non-tumor tissues) were sourced from the TCGA database.

### Dataset of meQTLs in lung tissues and blood samples

One of the datasets, “GTEx Lung meQTL (lung tissues),” was obtained from the Genotype-Tissue Expression (GTEx) project, comprising 223 lung tissue samples [24]. Another dataset, “Multi-racial normal meQTL (blood samples),” included blood samples from 3799 Europeans and 3195 South Asians [25].

### Selection of candidate meQTLs

Initially, differential methylation analysis (ChAMP package) was performed on both the in-house and TCGA methylation arrays to identify differentially methylated CpG sites with consistent findings across both datasets (*P*_FDR_ < 0.05). Next, the GTEx lung meQTL (lung tissue) and Multi-racial normal meQTL (blood sample) datasets were filtered for meQTLs with *P*_FDR_ < 0.05. The filtered meQTLs were then intersected with the differentially methylated CpG sites, allowing for the identification of LUAD-associated meQTLs correlated with CpG site methylation levels. Finally, candidate meQTLs were further refined based on the minor allele frequency (MAF) > 0.05 in the Chinese Han population (CHB) and *r*^*2*^ < 0.80 in linkage disequilibrium (LD) analysis.

### Study population for susceptibility analysis

A case-control study was conducted to examine the relationship between these candidate cis-meQTLs and LUAD risk. Study participants were drawn from the Genotype and Phenotype Database (dbGAP) *via* the Female Lung Cancer Consortium in Asia GWAS (accession number phs000716.v1.p1), comprising 3453 non-smoking LUAD cases and 3710 healthy controls. The specific analysis method for candidate meQTLs and LUAD risk can be found in the supplementary information.

### Study participants and sample details for immunohistochemical analysis

The 69 LUAD samples used for immunohistochemical analysis and corresponding imaging data were sourced from patients admitted to the First Affiliated Hospital of Nanjing Medical University, Hai’an People’s Hospital, and Tongzhou People’s Hospital. Inclusion criteria for the radiomics study included: (1) pathologically confirmed LUAD; (2) availability of dual-source CT imaging data collected prior to radiotherapy, chemotherapy, or surgery; and (3) complete prognostic data.

Detailed clinical data for these samples are presented in Supplementary Table 1. Informed consent was obtained from all participants, and the study protocol received approval from both the Medical Ethics Committee of Nantong University (Approval number: 2022-2) and the Ethics Committee of Nanjing Medical University (Approval number: IACUC-2206030). All methods were performed in accordance with the relevant guidelines and regulations.

### DNA/RNA sample processing

Procedures for DNA/RNA extraction, whole-genome DNA methylation detection, and qRT-PCR are provided in the supplementary information, with qRT-PCR primer sequences detailed in Supplementary Table 2. Target gene expression levels were calculated using the 2^-ΔΔCT^ method, with β-actin serving as the reference gene.

### Cell culture

The cell lines employed in this study-H1975, PC9, SPCA-1, and HEK293T-were purchased from the American Type Culture Collection (ATCC). Detailed culture conditions are described in the supplementary information.

### Demethylation with 5-Aza agent treatment

H1975, PC9, and SPCA-1 cells were seeded into six-well plates and treated with varying concentrations of 5-Aza-2’-deoxycytidine (5-Aza) at 0 (DMSO control), 2.5 μM, 5 μM, 7.5 μM, 10 μM, and 12.5 μM. Treatments were administered every other day for a total of three times, and on the sixth day, cells were harvested to obtain six sets of DNA and RNA per cell line.

Bisulfite sequencing PCR (BSP) was used to assess DNA methylation levels, and monoclonal sequencing was performed by Sangon Biotech (Shanghai) Co., Ltd., with results analyzed using DNAMAN software, version 9.0.

### Generation of overexpression cell lines

Lentiviral plasmids and overexpression vectors used in the study were procured from Corues Biotechnology (Nanjing, China). Based on the identified meQTL, stable cell lines overexpressing the *LRRC2* gene (Lv-*LRRC2*) and control lines with an empty vector (Lv-NC) were generated through lentiviral packaging. Fluorescence and qRT-PCR confirmed the expression levels in these cell lines. The steps for packaging lentivirus in HEK293T cells are provided in the supplementary information.

### Cell proliferation assay and transwell migration assay

Cell proliferation and migration assays were conducted using the overexpression cell lines, with detailed experimental protocols provided in the supplementary information.

### Tumor xenograft model

Animal experiments were approved by the Institutional Animal Care and Use Committee of Nantong University (Approval number: S20220224-006). Male BALB/c mice (4–5 weeks old) were obtained from GemPharmatech Co., Ltd. (Nanjing, China) and housed under specific-pathogen-free (SPF) conditions. The mice were randomly divided into two groups (*N* = 8 per group). H1975 cells expressing either Lv-NC or Lv-*LRRC2* (5 × 10^6^ cells in 100 μL) were subcutaneously injected into the right axilla of the mice. Tumor growth was measured regularly using calipers, and tumor volume was calculated with the formula: L (length) × W (width)^2^ × 2^−1^ at specified intervals. When tumors reached a volume of approximately 1000 mm³, the experiment was concluded, and all mice were euthanized. The tumors were photographed, weighed, and frozen for further analysis.

### Pathway enrichment analysis and risk analysis

Single-gene Gene Set Enrichment Analysis (GSEA) was performed on the target gene to carry out Gene Ontology (GO) and Kyoto Encyclopedia of Genes and Genomes (KEGG) pathway enrichment analyses. The Database for Annotation, Visualization, and Integrated Discovery (DAVID) were utilized for GO and KEGG pathway analysis of genes correlated with the target gene.

A two-sample Mendelian randomization analysis was conducted to assess the causal relationship between the candidate gene and LUAD. Our study utilizes cis-eQTLs, and the eQTL gene expression matrices (GTEx V8 cis-eQTL) were obtained from the GTEx database. The GWAS data for LUAD were sourced from GCST004744. The inverse variance weighted (IVW) method was used to determine the causal link between the candidate gene and LUAD [26, 27]. A meta-analysis was also performed to evaluate the association between candidate gene expression and survival in LUAD using the PrognoScan database, with further details provided in the supplementary information.

### Construction of a prognostic model using machine learning on related gene sets

This study incorporated samples from the TCGA-LUAD cohort (training set) and the GSE72094 cohort (validation set), using overall survival (OS) as the endpoint. A set of machine learning algorithms was employed to train the model based on genes identified as correlated with the target gene (|R| > 0.6, *P* < 0.05). The model with the highest mean concordance index (C-index) was selected as the optimal one [28]. Additionally, clinical data (age, gender, and tumor stage) were integrated into the model to construct a final LUAD mortality prognostic model, and its performance was assessed on the validation set using the area under the receiver operating characteristic (ROC) curve (AUC). Further methodological details are available in the supplementary information.

### Assessment of LRRC2 clinical and radiomics models in LUAD

The public data from the Cancer Imaging Archive (TCIA) LUAD-CT [29] were approved by the Institutional Review Board and included 45 samples, with 6 cases excluded due to unrecognizable lesions. In addition, we collected 69 LUAD samples locally and organized radiomics data from TCIA and local samples for subsequent analysis. Target delineation was performed independently by three licensed radiologists. We also performed immunohistochemical analysis of these 69 LUAD local samples.

Radiomics features were then extracted from TCIA, Consensus unsupervised cluster analysis was applied to classify the feature data, and Kaplan-Meier analysis was used to visualize survival differences between clusters. The cluster with the most significant survival disparity was selected for further analysis.

The risk indicators from the most significant cluster in the TCIA radiomics model were used to analyze the local samples. Local patients were stratified according to *LRRC2* immunohistochemical expression levels in LUAD tissues. Radiomics feature extraction and target delineation were also performed for these groups, and differences in risk indicators were compared between high and low *LRRC2* expression groups. Details of these procedures are provided in the supplementary information.

### Statistical analysis

Group comparisons were carried out using t-tests, Mann-Whitney U tests, or Wilcoxon tests, as appropriate. Correlation analysis was conducted using either the Pearson or Spearman method. Logistic regression analysis was employed to assess the association between meQTLs and LUAD risk, with adjustments for age. All bar graphs represent data from three or more independent experiments, as indicated in the figure legends. Statistical tests were two-sided, with a *P*-value < 0.05 considered statistically significant. All analyses were performed using R (version 4.3.1).

## Results

### Identification of differentially methylated CpG sites

**Fig. 1:**
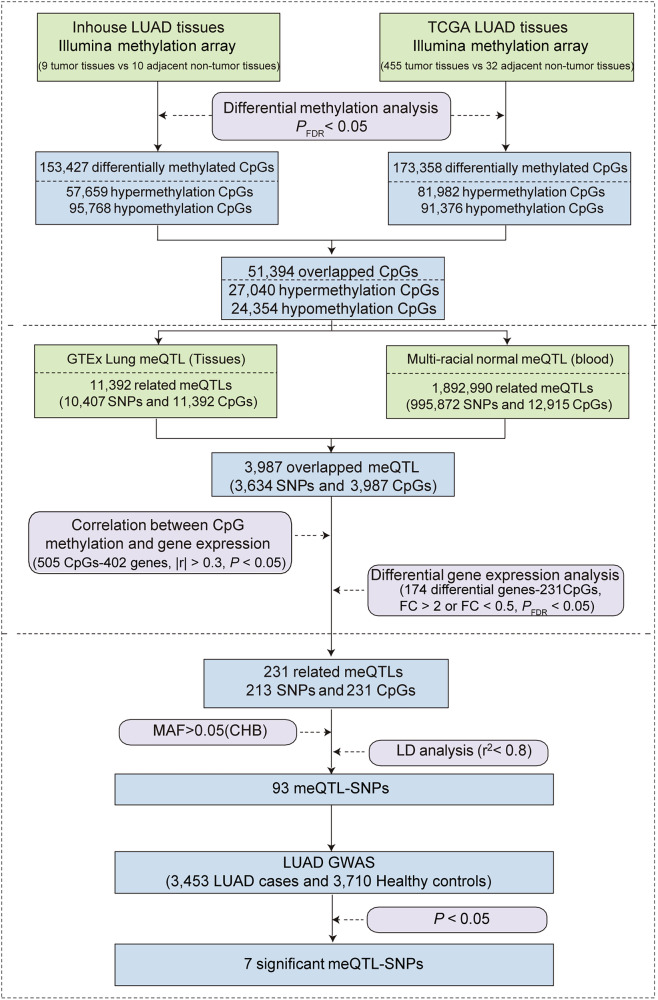
Schematic diagram of the multi-step analytical workflow for the identification of candidate meQTLs. A total of 51,394 overlapping CpG sites were identified, consisting of 27,040 hypermethylated and 24,354 hypomethylated sites. Cross-referenced with lung and blood meQTL datasets and correlation analysis resulting in the identification of 174 differentially expressed genes, corresponding to 231 CpGs and 213 SNPs. Finally, after applying minor allele frequency (MAF > 0.05) and linkage disequilibrium (LD, r^2^ < 0.8) filters, 93 meQTLs were selected. LUAD lung adenocarcinoma, meQTL methylation quantitative trait loci, SNPs single nucleotide polymorphisms, FC fold change, MAF minor allele frequency, CHB Chinese Han population in Beijing, LD linkage disequilibrium.

A total of 153,427 and 173,358 candidate differentially methylated CpG sites were selected from the in-house and TCGA datasets, respectively, using a threshold of *P*_FDR_ < 0.05. From this, 51,394 overlapping CpG sites were identified, consisting of 27,040 hypermethylated and 24,354 hypomethylated CpG sites (Fig. 1, Supplementary Fig. 1).

### Selection of candidate meQTLs

These 51,394 CpG sites were then cross-referenced with lung and blood meQTL datasets (11,392 CpGs in lung tissues and 12,915 CpGs in blood samples). This produced 3987 overlapping CpGs, corresponding to 3634 SNPs. Of these CpGs, 505 exhibited a significant correlation between methylation levels and the expression of 402 target genes (|r| > 0.3, *P* < 0.05; a target gene contains multiple CpG sites). To further refine the results, gene expression changes were filtered based on fold change (FC > 2 or FC < 0.5) and *P*_FDR_ < 0.05, leading to the identification of 174 differentially expressed genes, corresponding to 231 CpGs and 213 SNPs. Finally, after applying minor allele frequency (MAF > 0.05) and linkage disequilibrium (LD, *r*^*2*^ < 0.8) filters, 93 meQTLs were selected (Fig. 1).

### Susceptibility study of the association between the 93 candidate meQTLs and LUAD risk

Among the 93 identified meQTLs, 7 cis-meQTLs were significantly associated with altered LUAD risk in the GWAS database (*P* < 0.05) (Table 1, Table 2, Supplementary Table 3). Specifically, variant alleles of 3 meQTLs (rs12680375, rs328890, rs750373) were linked to an increased risk of LUAD (OR > 1), while variant alleles of the other 4 meQTLs (rs66719815, rs939408, rs3743281, rs2885221) were associated with a decreased risk (OR < 1). Furthermore, 4 of these SNPs (rs66719815, rs328890, rs750373, rs2885221) were correlated with increased methylation levels at their respective CpG sites (β > 0), whereas the remaining 3 SNPs (rs939408, rs12680375, rs3743281) were associated with decreased methylation at the corresponding CpG sites (β < 0).

**Table 1:** Detail information of the 7 identified meQTLs.

No.	CpG	SNP	Location	Gene	β direction (tissue)^a^	meQTL *P* -value (tissue)	β direction (blood)^a^	meQTL *P* value (blood)	Methylation level^b^	Gene expression level^c^	r^d^
1	cg19220282	rs66719815	chr2:65212470	*SLC1A4*	> 0	6.36E-15	> 0	9.93E-201	Low	High	−0.42
2	cg09596674	rs939408	chr3:46599079	*LRRC2*	< 0	1.75E-05	< 0	6.89E-08	High	Low	−0.32
3	cg10700718	rs12680375	chr8:1988076	*MYOM2*	< 0	0.02	< 0	1.38E-06	Low	Low	0.35
4	cg04065210	rs328890	chr7:35013449	*DPY19L1*	> 0	0.02	> 0	8.01E-39	Low	High	−0.48
5	cg04571833	rs750373	chr2:173833960	*RAPGEF4*	> 0	1.99E-14	> 0	3.80E-20	Low	Low	0.30
6	cg16110827	rs3743281	chr15:48056958	*SEMA6D*	< 0	2.24E-19	< 0	1.63E-213	Low	Low	0.49
7	cg03230154	rs2885221	chr19:22839387	*ZNF492*	> 0	2.01E-06	> 0	5.06E-47	High	Low	−0.46

^a^When β > 0, an increase in the predictor variable leads to an increase in the dependent variable, whereas when β < 0, an increase in the predictor variable leads to a decrease in the dependent variable.

^b^Represents the methylation level of the CpG site in the LUAD tumor tissue compared to adjacent non-tumor tissues.

^c^Represents the gene expression level in the LUAD tumor tissue compared to adjacent non-tumor tissues.

^d^Represents the correlation coefficient between the CpG site and the corresponding gene expression level.

**Table 2:** The associations between meQTLs and LUAD risk.

No.	CpG	SNP	Gene	Location	Alleles	Case	Control	MAF (Cases)	MAF (Controls)	OR (95% CI)^a^	*P*^a^
1	cg19220282	rs66719815	*SLC1A4*	chr2:65212470	T > C	2108/1183/162	2182/1331/197	0.218	0.232	0.92(0.85-0.99)	0.044
2	cg09596674	rs939408	*LRRC2*	chr3:46599079	C > A	2521/868/64	2623/1002/85	0.144	0.158	0.89(0.82-0.98)	0.019
3	cg10700718	rs12680375	*MYOM2*	chr8:1988076	G > A	2205/1108/140	2462/1125/123	0.201	0.185	1.11(1.02-1.21)	0.013
4	cg04065210	rs328890	*DPY19L1*	chr7:35013449	G > A	1483/1577/393	1704/1612/394	0.342	0.323	1.08(1.01-1.16)	0.027
5	cg04571833	rs750373	*RAPGEF4*	chr2:173833960	T > C	1035/1701/717	1178/1836/696	0.454	0.435	1.08(1.01-1.15)	0.025
6	cg16110827	rs3743281	*SEMA6D*	chr15:48056958	C > T	2365/987/101	2457/1130/123	0.172	0.185	0.91(0.84-0.99)	0.042
7	cg03230154	rs2885221	*ZNF492*	chr19:22839387	A > G	2527/854/72	2619/1010/81	0.145	0.158	0.90(0.82-0.99)	0.023

*LUAD* lung adenocarcinoma, *MAF* minor allele frequency, *OR* odds ratio, *CI* confidence intervals.

^a^Logistic regression analysis adjusted for age in the additive model.

### Checking the SNP-DNA methylation-gene expression-population susceptibility regulation pattern

**Fig. 2:**
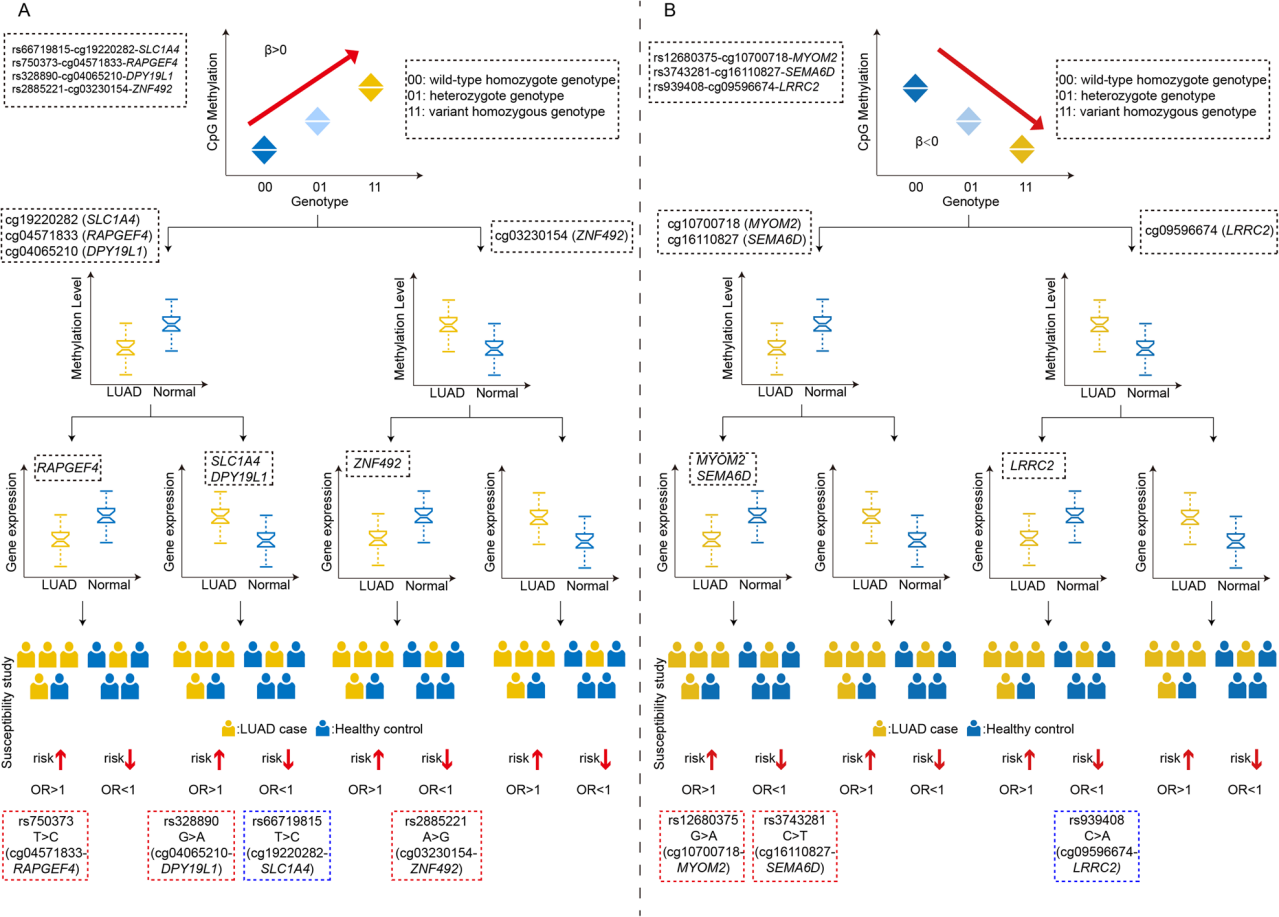
Potential pathogenesis linking meQTL to LUAD. This figure systematically illustrates the potential molecular mechanism by which meQTLs influence the development of LUAD through the regulation of DNA methylation. Specifically, we focused on the classical model of negative regulation, in which hypermethylation of CpG sites is associated with decreased target gene expression, and hypomethylation is associated with increased target gene expression. These methylation-mediated expression changes, driven by genetic variants at meQTL loci, may ultimately affect LUAD susceptibility. The blue border in the figure indicates verification through biological logic, while the red border represents failure to pass biological logic verification. **A** The case of β > 0 indicates that the mutant allele of a SNP is linked to higher methylation level of certain CpG site. **B** The case of β < 0 indicates that the mutant allele of a SNP is linked to lower methylation level of certain CpG site. LUAD lung adenocarcinoma, OR odds ratio.

The biological plausibility of the regulatory model linking SNPs, DNA methylation, gene expression, and LUAD susceptibility was further assessed for these 7 meQTLs (Fig. 2). For instance, in the case of the rs939408-cg09596674-*LRRC2* axis (Table 1 and Fig. 2), a β < 0 suggests that the mutant A allele of rs939408 is linked to lower methylation at the cg09596674 CpG site. Differential analysis showed that methylation levels of cg09596674 were elevated in LUAD tumor tissues compared to adjacent non-tumor tissues, while *LRRC2* expression was lower in tumor tissues. This implies that the mutant A allele of rs939408 is more prevalent in normal lung tissues, potentially reducing LUAD risk (OR < 1), as supported by the population susceptibility data (OR = 0.89, *P* = 0.019). Similarly, the rs66719815-cg19220282-*SLC1A4* axis also passed biological plausibility checks. But the remaining 5 meQTLs did not pass the biological plausibility validation.

### Gene expression and methylation level of selected meQTLs

**Fig. 3:**
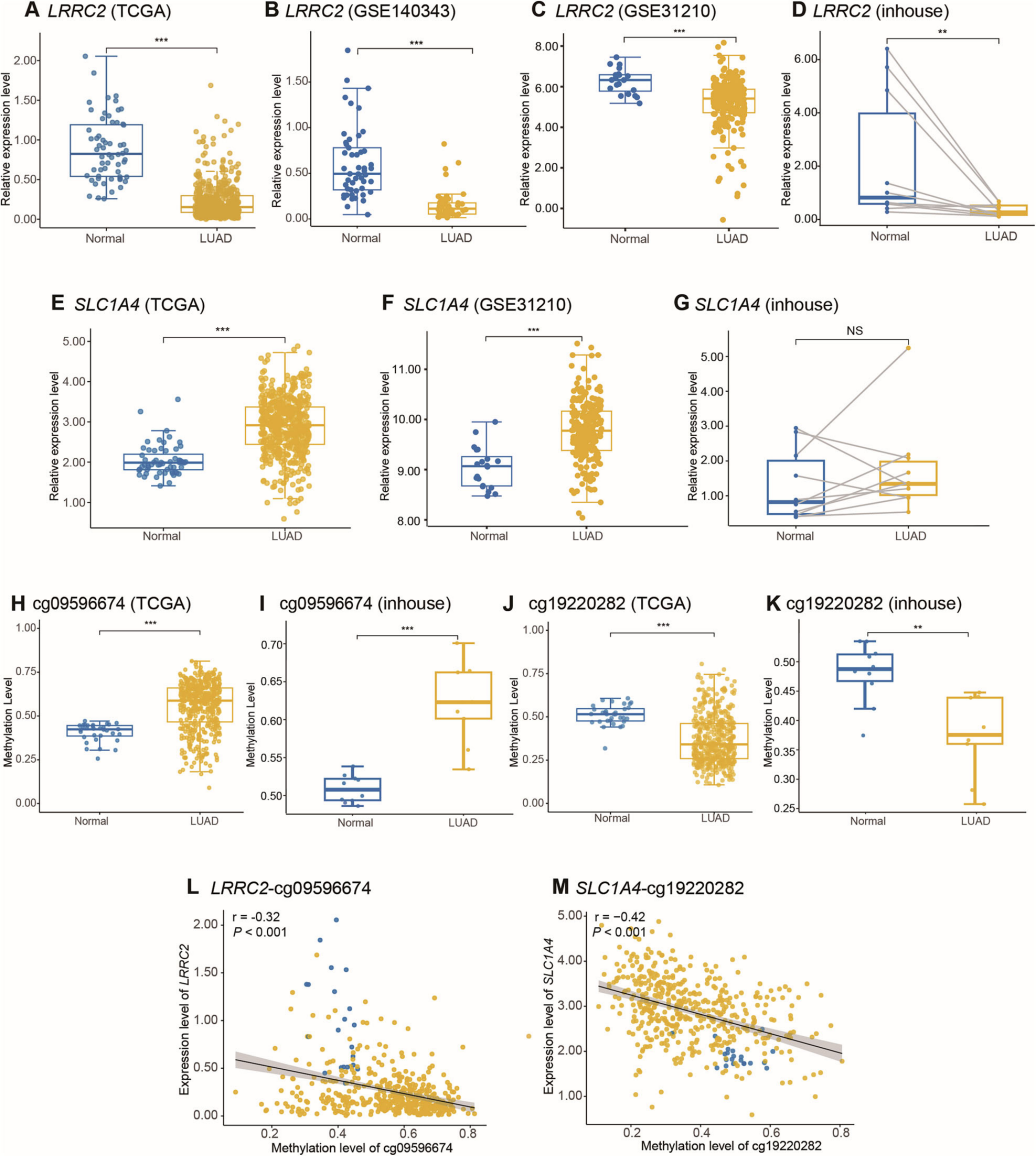
Expression and methylation levels of positive meQTL related genes and CpG sites in LUAD tumor tissues and adjacent non-tumor tissues. **A** *LRRC2* mRNA expression in the TCGA LUAD database (510 tumor tissues and 58 adjacent non-tumor tissues). **B** *LRRC2* mRNA expression in the GSE140343 dataset (49 tumor tissues and 49 adjacent non-tumor tissues). **C** *LRRC2* mRNA expression in the GSE31210 dataset (226 tumor tissues and 58 adjacent non-tumor tissues). **D** Comparison of *LRRC2* mRNA expression between LUAD tumor tissues and adjacent non-tumor tissues in in-house samples (10 tumor tissues and 10 adjacent non-tumor tissues). **E** *SLC1A4* mRNA expression in the TCGA LUAD database. **F** *SLC1A4* mRNA expression in the GSE31210 dataset. **G** Comparison of *SLC1A4* mRNA expression between LUAD tumor tissues and adjacent non-tumor tissues in in-house samples. **H** Methylation level of cg09596674 in LUAD tumor tissues and adjacent non-tumor tissues in the TCGA dataset (455 tumor tissues and 32 adjacent non-tumor tissues). **I** Methylation level of cg09596674 in in-house LUAD samples (9 tumor tissues and 10 adjacent non-tumor tissues). **J** Methylation level of cg19220282 in LUAD tumor tissues and adjacent non-tumor tissues in the TCGA dataset. **K** Methylation level of cg19220282 in in-house LUAD samples. **L** Correlation between cg09596674 methylation and *LRRC2* gene expression levels. **M** Correlation between cg19220282 methylation and *SLC1A4* gene expression levels. LUAD lung adenocarcinoma. Significance levels: ^*^P < 0.05, ^**^P < 0.01, ^***^P < 0.001.

In the TCGA dataset, *LRRC2* expression is significantly lower in LUAD tumor tissues compared to adjacent non-tumor tissues (Fig. 3A). This finding was further validated in the GSE140343 dataset (Chinese population) and GSE31210 dataset (Japanese population), both showing consistent results (Fig. 3B, C). Additionally, *LRRC2* expression in in-house LUAD tumor tissues was also lower than in adjacent non-tumor tissues (Fig. 3D). In subsequent expanded in-house LUAD tumor samples (*N* = 41), *LRRC2* expression remained significantly downregulated in tumor tissues (Supplementary Fig. 2).

For *SLC1A4*, its expression was significantly higher in LUAD tumor tissues compared to adjacent non-tumor tissues in the TCGA dataset (Fig. 3E), validation in the GSE31210 dataset confirmed the TCGA findings (Fig. 3F). However, *SLC1A4* data was unavailable in GSE140343, besides, in in-house samples, *SLC1A4* expression did not show statistical significance (Fig. 3G).

Regarding methylation levels, cg09596674 (*LRRC2*) exhibited higher methylation in both the TCGA and in-house LUAD tumor tissues compared to adjacent non-tumor tissues, while cg19220282 (*SLC1A4*) showed consistently lower methylation in LUAD tumor tissues (Fig. 3H–K).

### Results of demethylation with 5-Aza agent treatment

**Fig. 4:**
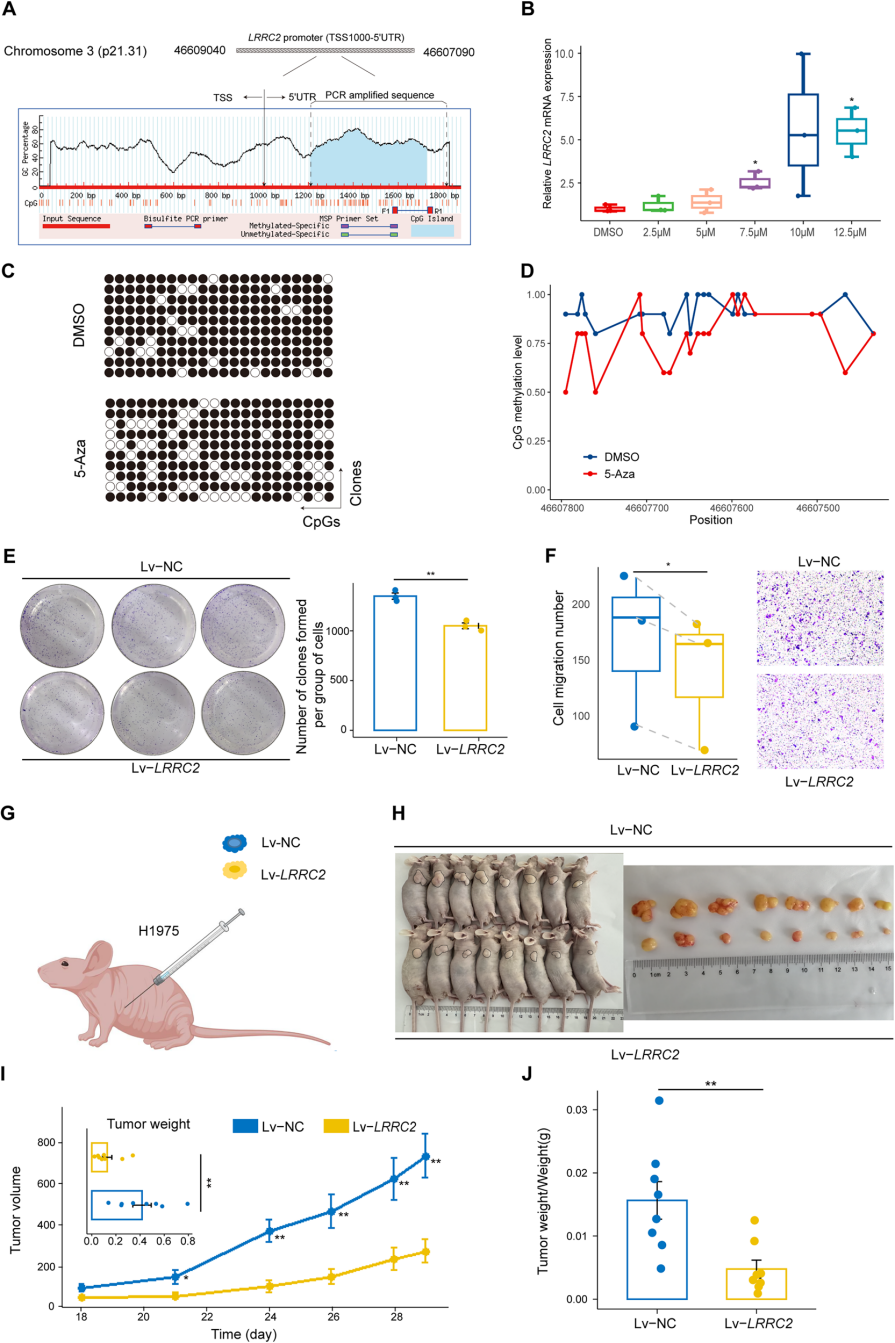
Effects of 5-Aza treatment and *LRRC2* on LUAD cell malignant phenotype and tumor growth. **A** Design of the amplified sequence for the *LRRC2* gene promoter CpG island used in the BSP experiment, highlighting the target region for methylation analysis. **B** Expression levels of *LRRC2* in demethylated H1975 cell lines treated with various concentrations of 5-Aza show a significant increase compared to the DMSO-treated control group. Results were showed as the mean ± standard deviation (*N* = 3). **C** BSP dot plot showing reduced methylation levels in the 5-Aza treatment group compared to the DMSO group. Empty dots indicate unmethylated CpG sites, while filled dots represent methylated CpG sites. **D** BSP line graph further confirming that the 5-Aza group exhibits lower methylation levels than the DMSO group. **E** Cell proliferation assay conducted on H1975 LUAD cells after transfection with Lv-*LRRC2* or control vector (Lv-NC). The results indicate that *LRRC2* overexpression significantly inhibits cell proliferation compared to the control group. Results were showed as the mean ± standard deviation (*N* = 3). **F** Transwell migration assay assessing the migratory capacity of H1975 cells following transfection with either Lv-*LRRC2* or Lv-NC. The number of migrated cells was quantified and statistically analyzed using a paired t-test, demonstrating that *LRRC2* overexpression markedly reduces H1975 cell migration. Each experiment was performed in triplicate (*N* = 3). **G** Schematic diagram of the xenograft tumor model used for in vivo studies. Nude mice were subcutaneously injected with H1975 cells transfected with either Lv-*LRRC2* or Lv-NC, followed by observation of tumor growth over time. **H** Macroscopic view of tumors in nude mice. **I** Quantitative comparison of tumor growth between the Lv-NC group (*N* = 8) and the Lv-*LRRC2* group (*N* = 8). Tumor volumes were measured regularly during the experiment and final tumor weights were recorded at the endpoint. Results are expressed as mean ± standard deviation, indicating that *LRRC2* overexpression significantly inhibits tumor growth in vivo. **J** Ratio of tumor weight to body weight at the endpoint of the xenograft experiment for mice in the Lv-NC (*N* = 8) and Lv-*LRRC2* (*N* = 8) groups. Data are shown as mean ± standard deviation, further supporting the tumor-suppressive role of *LRRC2* in vivo. 5-Aza 5-Aza-2’-deoxycytidine, BSP bisulfite sequencing PCR. Significance levels: ^*^P < 0.05, ^**^P < 0.01, ^***^P < 0.001.

In the TCGA dataset, methylation levels at CpG sites cg09596674 and cg19220282 were negatively correlated with the expression of *LRRC2* (r = −0.32, *P* < 0.001) and *SLC1A4* (r = −0.42, *P* < 0.001), respectively (Fig. 3L, M). Following 5-Aza demethylation treatment, *LRRC2* mRNA expression in the H1975 cell line increased with higher concentrations of 5-Aza, while methylation in the *LRRC2* promoter region was reduced (Fig. 4A–D). In contrast, *SLC1A4* gene expression remained statistically insignificant after 5-Aza treatment in H1975, PC9, and SPCA-1 cells (Supplementary Fig. 3A–F).

Given the consistent and statistically significant expression of *LRRC2* across the TCGA, GSE140343, and GSE31210 datasets and in-house samples, along with the upregulation observed in the 5-Aza demethylation experiment, *LRRC2*, associated with the meQTL rs939408, was selected for further mechanistic research.

### In-depth analysis of differential LRRC2 expression between tumor and normal lung tissues

Using TCGA-LUAD (513 tumor tissues and 59 adjacent non-tumor tissues) and GTEx normal lung tissues (288 normal lung tissues, which were obtained from healthy deceased donors through a rapid autopsy protocol) datasets. Our systematic comparison shows that *LRRC2* has a consistent and statistically inhibitory effect in LUAD, and the expression in tumor tissues is significantly lower than that in adjacent non-tumor tissues (*P* < 0.0001), normal lung tissues (*P* < 0.0001), and the combined normal tissues (adjacent non-tumor tissues + normal lung tissues, *P* < 0.0001) (Supplementary Fig. 4).

### Effect of LRRC2 overexpression on the malignant phenotype of LUAD in vivo and in vitro

After stably transfecting H1975 cells with the Lv-*LRRC2* and Lv-NC plasmids, the expression level in the Lv-*LRRC2* group was significantly higher than that in the Lv-NC group (Supplementary Fig. 5A, B). Further investigations revealed that *LRRC2* overexpression effectively inhibited both cell proliferation and migration in H1975 cells (Fig. 4E, F). To assess the impact of *LRRC2* on tumor growth in vivo, H1975 cells transfected with Lv-NC and Lv-*LRRC2* were injected into nude mice (Fig. 4G). Tumor volume in the Lv-*LRRC2* group increased at a slower rate compared to the Lv-NC group. On the 29th day, after euthanizing the mice, the tumor weight in the Lv-*LRRC2* group was found to be significantly lower than in the Lv-NC group (Fig. 4H–J), indicating that *LRRC2* overexpression significantly inhibits tumor growth.

### Gene enrichment analysis and survival prognosis results of LRRC2

**Fig. 5:**
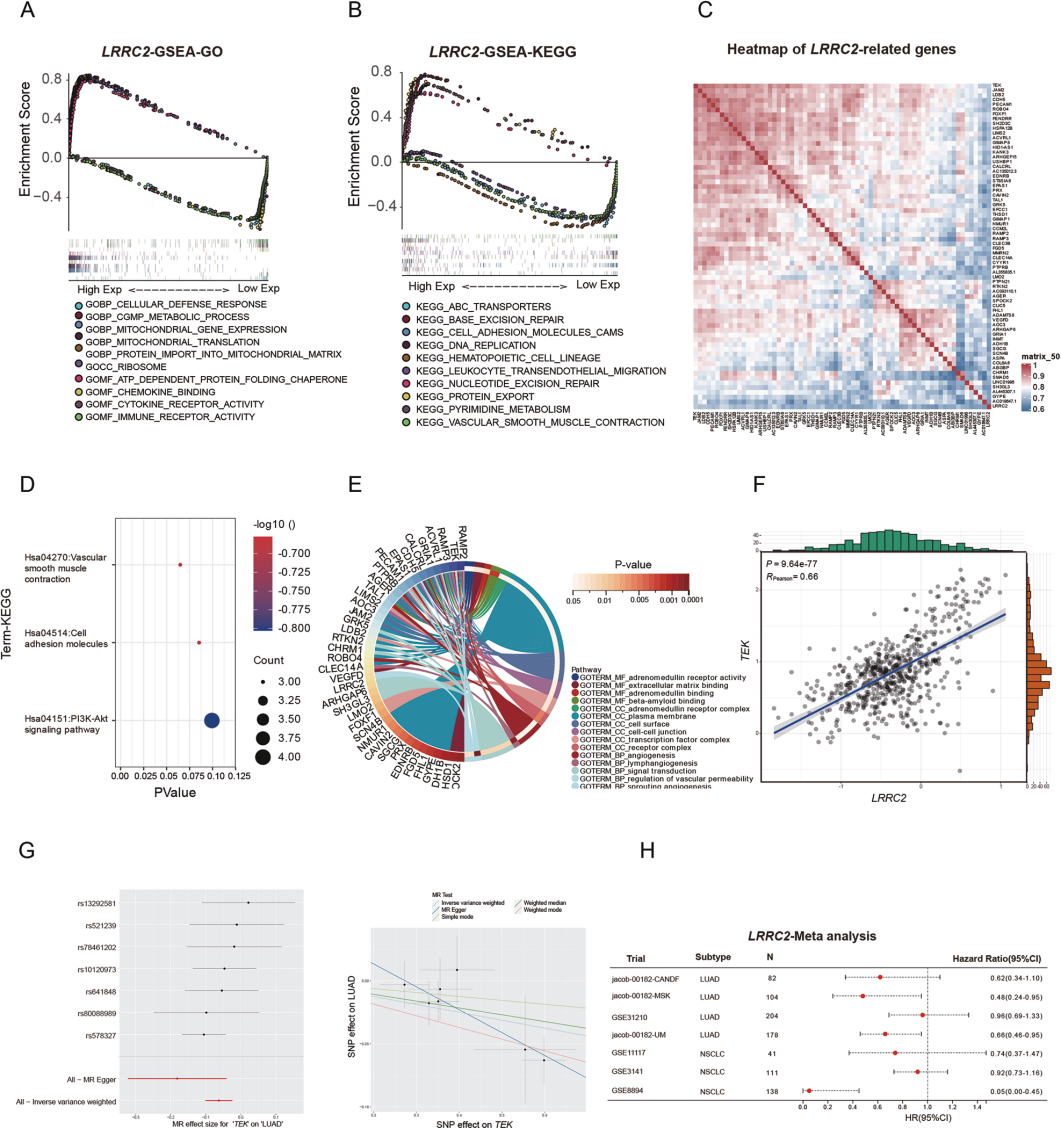
Comprehensive analysis of *LRRC2* expression using public databases. **A** GSEA of GO terms associated with *LRRC2* expression levels in LUAD, highlighting biological processes potentially regulated by *LRRC2*. **B** GSEA of KEGG pathways linked to *LRRC2* expression, identifying signaling pathways potentially influenced by *LRRC2* activity in LUAD. **C** Heatmap visualization of genes significantly correlated with *LRRC2* expression. Each row represents a gene, and the color intensity reflects the degree of correlation across samples. **D** KEGG pathway enrichment analysis of genes positively or negatively associated with *LRRC2*, revealing key molecular pathways in which *LRRC2*-related genes may be involved. **E** GO term enrichment analysis of *LRRC2*-associated genes, providing functional annotation in terms of biological processes, cellular components, and molecular functions enriched among *LRRC2*-correlated gene sets. **F** Scatter plot illustrating the correlation between *LRRC2* and *TEK* expression levels, showing a strong positive association that suggests potential co-regulation or interaction. **G** Mendelian randomization analysis validating the causal relationship between *TEK*, a gene highly correlated with *LRRC2*, and LUAD. **H** Meta-analysis of *LRRC2* expression data across multiple datasets. LUAD lung adenocarcinoma, GSEA Gene Set Enrichment Analysis, GO Gene Ontology, KEGG Kyoto Encyclopedia of Genes and Genomes.

GSEA of *LRRC2* revealed significant enrichment in pathways related to mitochondrial function (Fig. 5A) and ATP-binding cassette (ABC) transporters (Fig. 5B). Correlated genes (|R | > 0.6, *P* < 0.05) with *LRRC2* (Fig. 5C) were primarily associated with the PI3K-AKT signaling pathway and angiogenesis (Fig. 5D, E). Notably, *TEK* exhibited a strong correlation with *LRRC2* (R = 0.66, *P* = 9.64 × 10^−77^) (Fig. 5F). To validate a potential causal relationship between *TEK* and LUAD, this study analyzed SNPs with cis-expression quantitative trait loci (cis-eQTLs) in *TEK*, including 7 SNPs in the analysis (Fig. 5G). A meta-analysis of *LRRC2* expression data suggested that *LRRC2* may serve as a protective factor, positively influencing survival in NSCLC (Fig. 5H).

### LRRC2-based machine learning and radiomics models

**Fig. 6:**
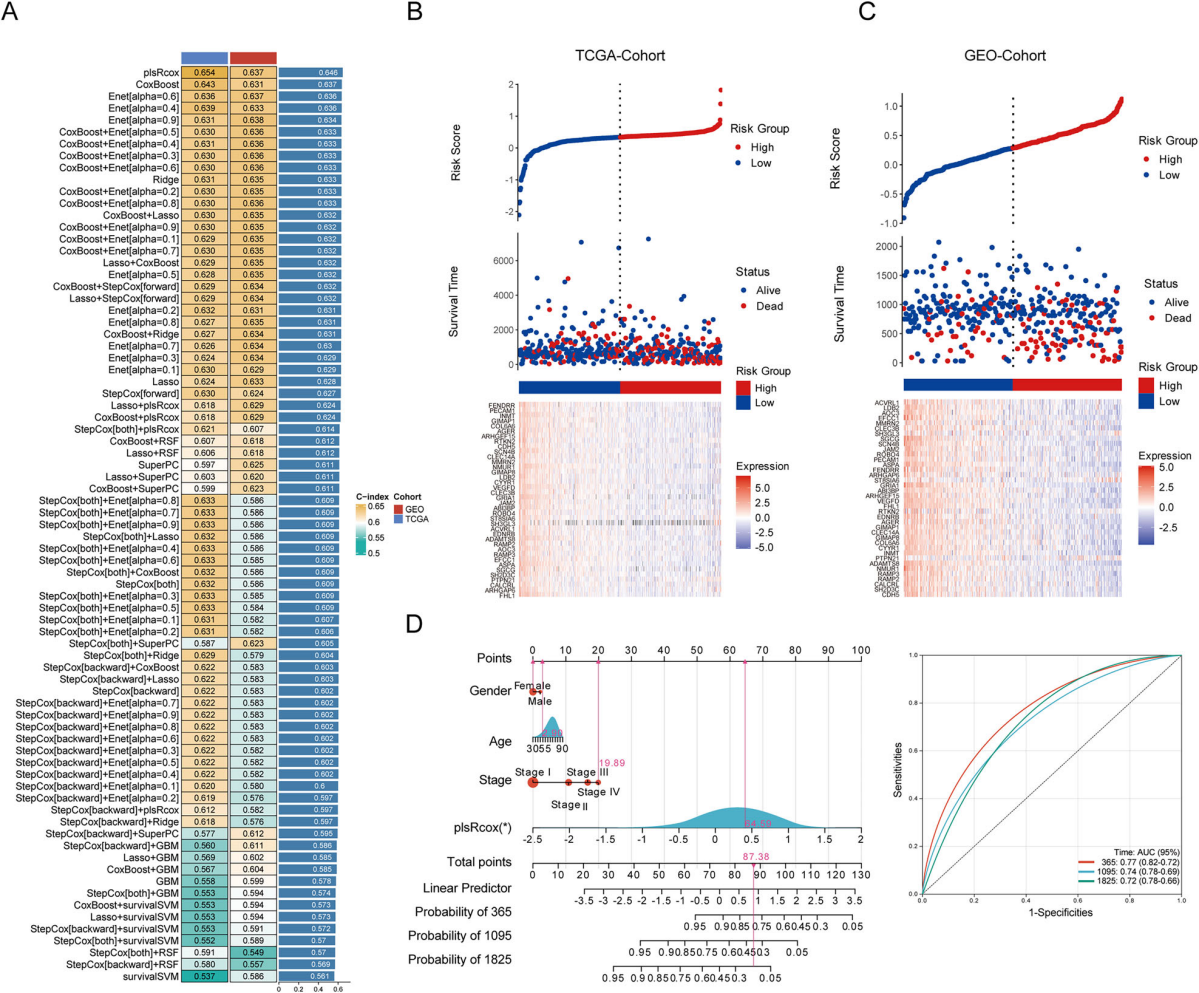
Development and validation of *LRRC2*-centered machine learning prognostic models in LUAD. **A** Comparative analysis of various machine learning algorithms used to construct prognostic models centered around *LRRC2*. **B**, **C** Risk score calculation using the plsRcox model for both the TCGA-LUAD (training set) and GSE72094 (validation set) cohorts. The heatmap illustrates the relative influence of the 38 genes included in the model. **D** Nomogram integrating clinical data with the *LRRC2*-centered model for prognostic predictions. ROC curves validate the predictive performance for 1-, 3-, and 5-year OS. ROC Receiver Operating Characteristic, OS overall survival.

After generating multiple prognostic models, the plsRcox-based model was selected as the optimal choice due to its highest average C-index of 0.646 (Fig. 6A). This model incorporated 38 genes, with their relative contributions shown in Fig. 6B. Using this model, risk scores were calculated for each patient across various cohorts (Fig. 6B, C). A nomogram was constructed, and the AUC values for 1-, 3-, and 5-year OS in the training set were 0.77, 0.74, and 0.72, respectively (Fig. 6D).

**Fig. 7:**
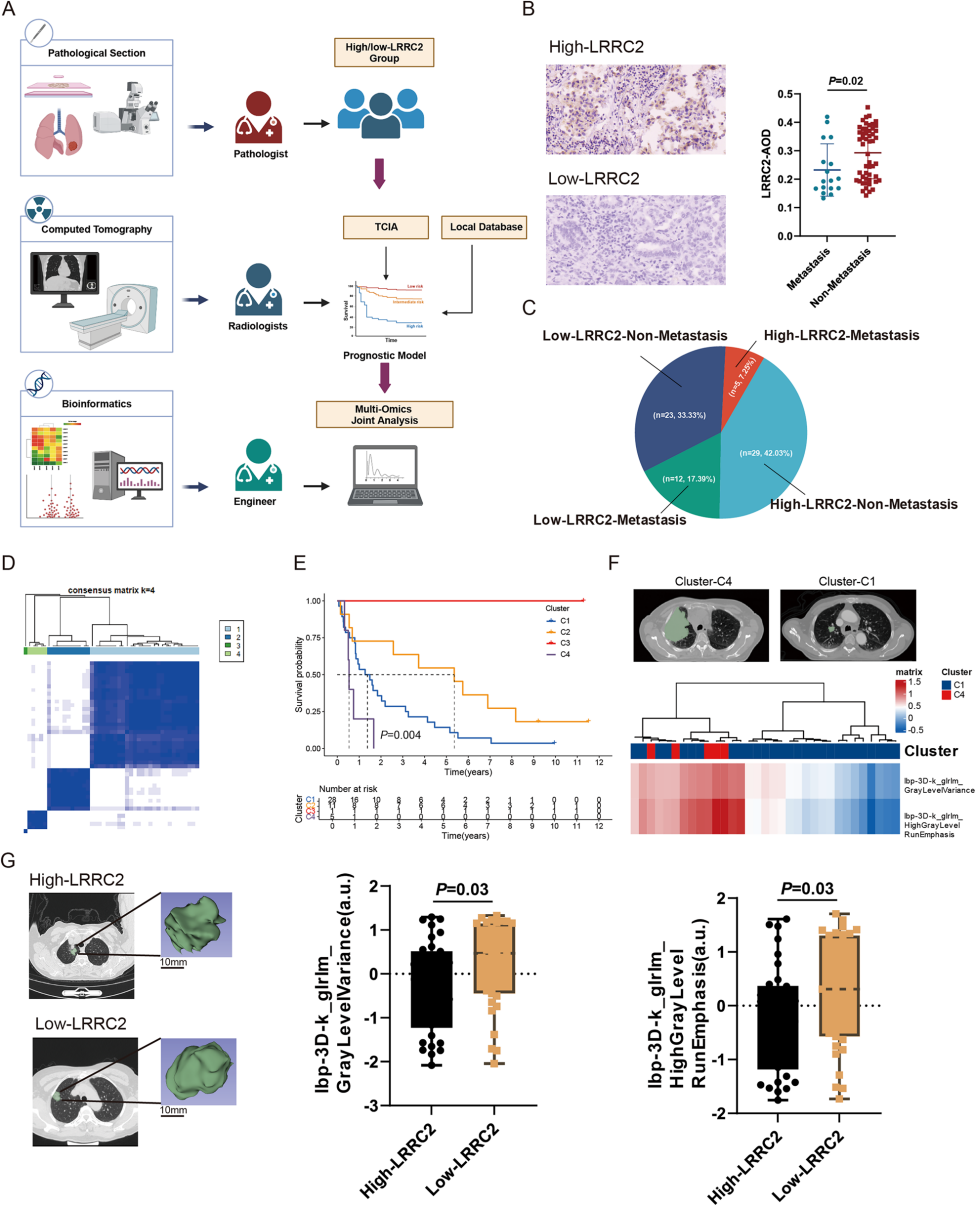
Radiomics model validation of *LRRC2* clinical characteristics. **A** Schematic representation of the radiomics model construction process. Target delineation was performed independently by three licensed radiologists for TCIA samples and local samples. **B** Immunohistochemical staining results of *LRRC2* protein in LUAD tumor tissue samples from local patients (*N* = 69). **C** Pie chart depicting the proportion of distant metastasis in relation to *LRRC2* expression levels in patients with LUAD. **D** Cluster analysis of LUAD features extracted from the TCIA radiomics public database, with mortality as the endpoint. **E** Comparison of survival times across the four identified clusters. **F** Heatmap displaying the two key imaging features, lbp-3D-k_glrlm_GrayLevelVariance and lbp-3D-k_glrlm_HighGrayLevelRunEmphasis, between the C1 and C4 clusters from the TICA database. Representative target area images from both clusters (C4 and C1) are shown. **G** Representative lung CT images and corresponding 3D tumor reconstructions from samples with high and low *LRRC2* expression in the local LUAD dataset. Comparative analysis of lbp-3D-k_glrlm_GrayLevelVariance and lbp-3D-k_glrlm_HighGrayLevelRunEmphasis between different *LRRC2* expression groups is also presented. LUAD lung adenocarcinoma.

Figure 7A outlines the radiomics model construction process. Based on immunohistochemical staining of local tissue samples, patients were divided into high and low *LRRC2* expression groups using median values. Combined with clinical follow-up data, low *LRRC2* expression was significantly associated with metastasis in patients with LUAD (*P* = 0.02) (Fig. 7B, C). Subsequently, 1701 CT imaging features were extracted from LUAD cases in public databases using PyRadiomics. Unsupervised clustering identified four sample groups, with C1 and C4 showing the most significant difference (*P* = 0.004), and C4 demonstrating the poorest survival prognosis (Fig. 7D, E). Further analysis of the C1 and C4 groups highlighted two key poorer survival-related imaging features: lbp-3D-k_glrlm_GrayLevelVariance and lbp-3D-k_glrlm_HighGrayLevelRunEmphasis (the specific meaning of these features is provided in the supplementary information). A heatmap illustrates the distribution of these features between the two groups, revealing their association with mortality. Representative target area images from the C4 and C1 groups are also provided (Fig. 7F). Figure 7G displays representative CT images and corresponding 3D tumor reconstructions for patients with high and low *LRRC2* expression. Combining the mortality-related imaging features with *LRRC2* expression for analysis, it was found that low *LRRC2* expression correlated with higher levels of lbp-3D-k_glrlm_GrayLevelVariance (*P* = 0.03) and lbp-3D-k_glrlm_HighGrayLevelRunEmphasis (*P* = 0.03), indicating that decreased *LRRC2* expression increases mortality risk in patients with LUAD.

## Discussion

In this study, seven meQTLs associated with non-smoking LUAD risk were identified through DNA methylation array datasets, meQTL datasets, and validation using the LUAD GWAS. Among these, the meQTL rs939408 and its corresponding gene *LRRC2* were further validated experimentally, revealing that methylation changes at the CpG site (cg09596674), linked to rs939408, may influence *LRRC2* expression. Overexpression of *LRRC2* was shown to inhibit the malignant phenotype of LUAD cell lines and suppress tumor growth in vivo. Additionally, reduced *LRRC2* expression was correlated with metastasis and poorer survival-related imaging features. This study provides insights into the etiology of LUAD, highlighting the molecular genetic and epigenetic mechanisms involved.

*LRRC2*, a member of the leucine-rich repeat-containing (LRRC) protein family, is increasingly recognized as a potential target for tumor diagnosis and therapy. Variations in the expression of LRRC superfamily members have been observed across different malignancies [30]. *LRRC2* is localized in human mitochondria and is transcriptionally regulated by the mitochondrial master regulator Pgc-1α. Enrichment analysis in this study identified significant involvement in mitochondrial function pathways. Mitochondrial dysfunction is a hallmark of several diseases, including Alzheimer’s and cancer [31]. A close relative of *LRRC2* is Ras Suppressor Protein 1 (RSP-1), which plays a role in the Ras signaling pathway. Similar to RSP-1, *LRRC2* can inhibit the conversion of v-ras, a key oncogene in the Ras gene family, in vitro [32]. Given the critical role of aberrant Ras signaling in tumor progression, targeting this pathway alongside autophagy has been shown to significantly inhibit tumor proliferation and promote apoptosis [33]. Collectively, these findings suggest that *LRRC2* is closely linked to tumorigenesis and may have tumor-suppressive functions.

To further investigate the functional role of *LRRC2* in normal lung tissue, we stratified GTEx normal lung tissue samples (*N* = 288) into *LRRC2* high-expression (top 25%) and low-expression (bottom 25%) groups based on *LRRC2* expression levels. Differential gene expression analysis identified 3837 significantly upregulated genes and 114 downregulated genes (*P*_FDR_ < 0.05). Pathway enrichment analysis revealed *LRRC2*’s potential involvement in 189 significantly enriched pathways (*P* < 0.05), including lung-specific processes such as Lung alveolus development, Lung development, and Respiratory tube development (Supplementary Fig. 6), suggesting a putative role for *LRRC2* in maintaining the normal physiological processes of the lungs.

Several studies have investigated the impact of SNP mutations on LUAD risk. Dai et al. [8] identified 19 susceptibility loci associated with NSCLC, including SNP loci related to LUAD risk. Yu et al. [34] discovered a novel regQTL-SNP (rs3768617), which may influence lung cancer risk by modulating the expression of miRNA-548b-3p and *LAMC1*. Xu et al. [21] identified three apaQTL/eQTL-SNPs (rs10452178, rs11714045, and rs277646) that could affect LUAD susceptibility. These studies proposed several pathogenic mechanisms, positioning the identified SNPs as potential biomarkers for early LUAD diagnosis. However, many aspects remain unexplored. This study shifts the focus to meQTLs, aiming to identify novel SNPs involved in DNA methylation-related pathogenic mechanisms of LUAD.

Research has shown that genetic variations can subtly influence DNA methylation, thereby modulating mRNA expression [35]. In cancer studies, Xie et al. identified variants rs10514231 and rs1864182 as being associated with higher methylation levels at cg17942617, resulting in elevated *ATG10* expression and reduced survival [36]. Similarly, our findings reveal that the meQTL rs939408 is negatively correlated with methylation at CpG site cg09596674. Experimental results further demonstrate that methylation at this CpG site negatively affects *LRRC2* gene expression. Promoter hypermethylation commonly suppresses gene expression, especially in tumor suppressor genes [37]. This is consistent with the behavior of cg09596674 and *LRRC2*. Besides, our integrative analysis combining methylation profiling, transcription factor (TF) prediction, and 3D chromatin context demonstrates that cg09596674 and rs939408 are embedded within a structurally and functionally unified regulatory framework (Supplementary Fig. 7). Their joint positioning within the same Topologically Associating Domain (TAD) provides a plausible mechanistic link for their observed impact on *LRRC2* transcription, reinforcing the biological significance of this meQTL.

**Fig. 8:**
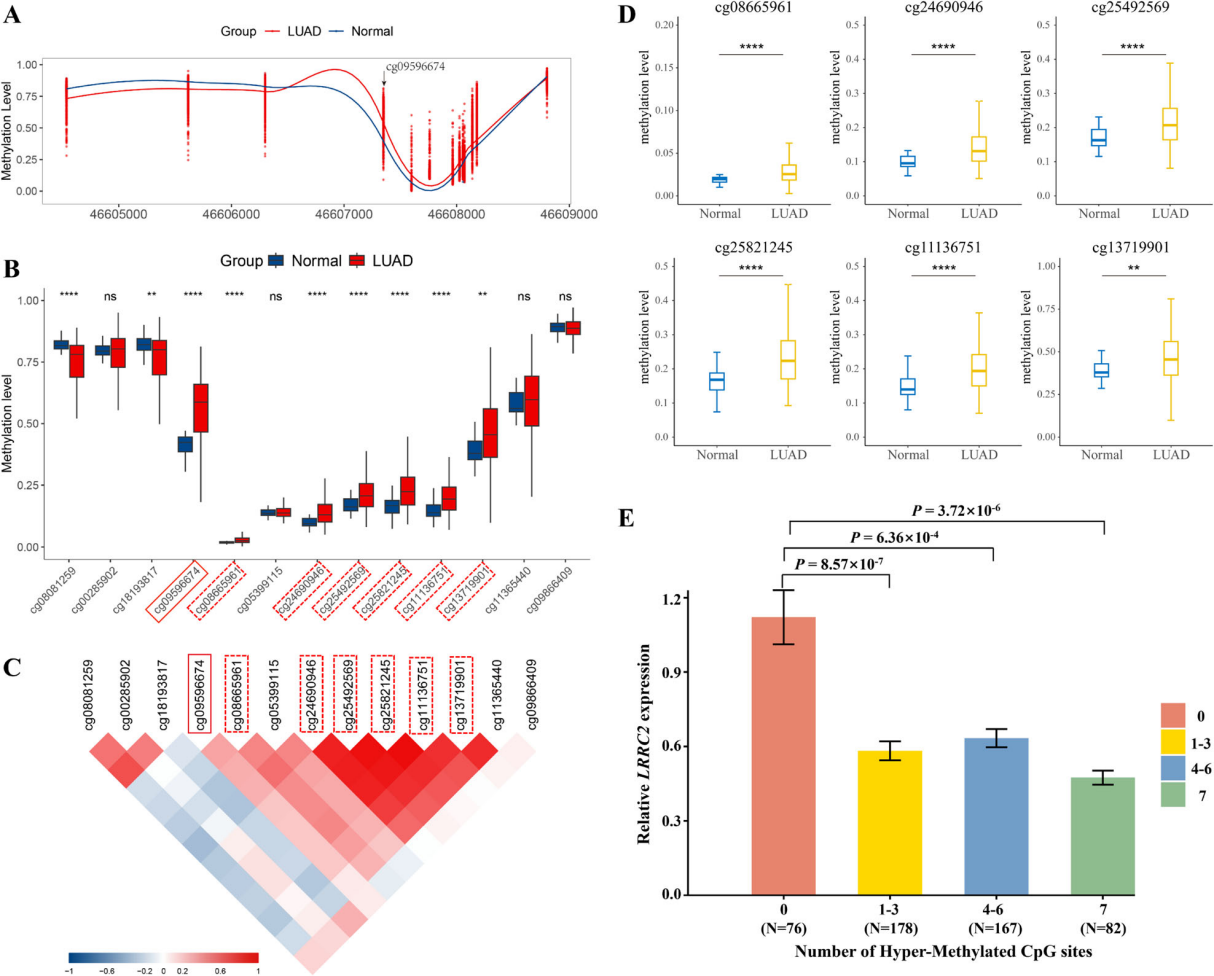
Integrated analysis of DNA methylation at cg09596674 and adjacent CpG sites, and their joint effect on *LRRC2* gene expression in LUAD. **A** Genomic localization of CpG Sites within the cg09596674-associated CpG island in *LRRC2*. **B** Differential methylation levels of CpG sites within the cg09596674-associated CpG island, among 13 CpG sites, 7-including cg09596674-exhibited significantly higher methylation levels in LUAD tumor tissues, while two showed hypomethylation, and four had no significant difference. **C** Correlation matrix of methylation levels among CpG Sites within the CpG Island, six CpG sites (cg08665961, cg24690946, cg25492569, cg25821245, cg11136751, and cg13719901) were co-hypermethylated with cg09596674. **D** Differential methylation analysis of 6 CpG Sites, which were significantly hypermethylated in LUAD tumor tissues. **E** Joint effect of CpG site hypermethylation on *LRRC2* gene expression in LUAD. LUAD lung adenocarcinoma.

Cg09596674 is located within the CpG island in the promoter region of the *LRRC2* gene, at position Chr3:46607350. In LUAD, CpG sites within the same CpG island (cg08665961, cg24690946, cg25492569, cg25821245, cg11136751, cg13719901) also exhibit hypermethylation and can be considered co-methylated sites. In view of the modest or small effect of single CpG site, we put the above identified cg09596674 as well as six CpG sites (cg08665961, cg24690946, cg25492569, cg25821245, cg11136751, and cg13719901) that were co-hypermethylated with cg09596674 (total seven CpG sites) together to assess the joint effect. We found that the more hyper-methylated CpG sites the subjects carried, the lower expression level of *LRRC2* they have, suggesting an allele-dosage effect (Fig. 8). Individuals with ‘1–3’ hyper-methylated CpG sites had a lower expression level of *LRRC2* compared with those having ‘0’ hyper-methylated CpG site (*P* = 8.57 × 10^−7^), similar trend was also found in individuals with ‘4–6’ hypermethylated CpG sites (*P* = 6.36 × 10^−4^). The lower expression level of *LRRC2* was more evident among subjects having ‘7’ hyper-methylated CpG sites (*P* = 3.72 × 10^−6^). Although these CpG sites are not regulated by SNPs, we cannot exclude the possibility that they may regulate related genes through other mechanisms. Future research holds the potential to shed light on the mechanisms and broader implications of this phenomenon, offering deeper insights into its nature. Functional experiments confirmed *LRRC2* as a tumor suppressor gene, with its high expression inhibiting malignant phenotype progression. SNP-induced genetic variation contributes to individual differences in DNA methylation [38]. A deeper understanding of the interaction between SNPs, DNA methylation, and gene expression may offer insights into disease mechanisms and support the development of personalized therapies.

This study has several strengths. Firstly, our validation results consistently showed that the identified rs939408-cg09596674 relationship was statistically significant across five independent databases by conducting cross-validation using six ethnically diverse meQTL datasets from Sino-mQTL (https://www.biosino.org/sinomqtl/browse) (Supplementary Fig. 8), This consistent replication across ethnically and genetically diverse populations provides strong support for the robustness and trans-ethnic reproducibility of the association. Secondly, by utilizing the latest publicly available meQTL database, the study combined data from lung tissues and blood samples to ensure consistent differential expression across sources, making the findings more robust, furthermore, blood samples are more accessible due to their minimal invasiveness and higher patient acceptability, which enhances their potential for broader application in the future. The identification of consistent meQTLs in both blood and lung tissue may further advance their utility in clinical research. Thirdly, rs939408 consistently showed statistically significant associations with lung cancer risk across two independent non-smoking lung cancer GWAS datasets of European ancestry from the IEU OpenGWAS project (https://gwas.mrcieu.ac.uk/). This consistency across different European GWAS strengthens the robustness of our findings and suggests that the association may extend to a broader range of ethnic groups, thereby improving the generalizability of our results. Fourthly, the use of machine learning and radiomics models, which integrated target genes with clinical data, enhances the study’s translational relevance and potential for clinical application.

Nevertheless, several limitations should be addressed. Firstly, given the currently limited follow-up duration for our local LUAD patients, we used metastasis as a surrogate endpoint to assess prognostic relevance. While metastasis is strongly associated with death (poor survival) both clinically and statistically, and accounts for the majority of cancer-related deaths, it cannot be equated directly with mortality. Therefore, our prospective local cohort study is ongoing, with continued follow-up and planned enrollment expansion. This may further enhance statistical power to definitively validate our findings. Secondly, when evaluating the association between target genes and LUAD progression, the retrospective nature of the TCGA-derived training cohort inherently limits tracking of the disease continuum from onset to progression, however, our validation local cohort employs a prospective design with systematic follow-up, which may further facilitate comprehensive evaluation of target genes’ predictive role in LUAD progression. Thirdly, through stratified and interaction analyses, we found that environmental factors, particularly smoking, did not significantly alter the negative association between cg09596674 methylation and *LRRC2* gene expression (Supplementary Figs. 9–11). Further investigation with larger samples or different methodologies might be warranted to explore potential modifying effects more comprehensively.

## Conclusion

In conclusion, mutations in 7 meQTLs were identified as potential factors influencing non-smoking LUAD risk. Notably, the meQTL rs939408 may affect *LRRC2* expression by modulating DNA methylation, thereby impacting non-smoking LUAD susceptibility. These findings highlight the link between meQTLs and LUAD risk, offering valuable insights for further investigation into the molecular mechanisms underlying LUAD carcinogenesis.

## Supplementary information

Supplementary Information

## Data Availability

The public LUAD data used in this study, obtained from databases such as TCGA, GEO, TCIA, as well as the meQTL dataset and case-control GWAS, are available on their respective websites. The URLs and login credentials can be found in the Supplementary information. All other datasets and code generated during the current study are available from the corresponding author on reasonable request.
