# Supplementary material for: Integrated genetic and epigenetic analysis identifies that rs939408 affects non-smoking lung adenocarcinoma risk by modulating the DNA methylation of LRRC2
Source: Cell Death Dis. 2025 Nov 17;16(1):836. doi: 10.1038/s41419-025-08163-1 (PMC12624109; doi:10.1038/s41419-025-08163-1)
Supplement: Supplementary file 1 — Supplementary Information [file 41419_2025_8163_MOESM1_ESM.docx]

**Integrated genetic and epigenetic analysis identifies that rs939408 affects non-smoking lung adenocarcinoma risk by modulating the DNA methylation of *LRRC2***

**Lei Zhang^1 *^, Zhenyu Li ^1 *^, Yanchi Wang^2 *^, Mingjiong Zhang^3 *^, Haoyan Chen^3 *^, Yifan Cheng^3^, Qiong Chen ^1^, Baosheng Cui^4^, Jiahao Liu^1^, Haiyan Gong^4^, Rui Zhu^1^, Tian Tian^1^, Yan Zhang^1^, Shengguang Ding^5^, Yu Duan^3 #^, Shuangshuang Wu^3 #^, Minjie Chu^1 #^**

# Supplementary Methods

***Study population for susceptibility analysis***

The SNP sites obtained from the previous screening steps will be grouped according to their chromosomal positions. For instance, chromosome 1 will form one group, and chromosome 2 will form another, with each group stored in a separate txt file. Data extraction will then be performed for each group individually. To run Plink and ensure the program is functioning properly, enter the following command:

plink --bfile chr1 --extract SNP.txt --recode --make-bed --out SNP-2.

Here, "SNP" refers to the custom file name. Next, input the following two commands for logistic regression, adjusting for the covariate:

plink --bfile SNP-2 --keep covar_age_LUAD_ID.txt --recode --make-bed --out SNP-c

plink --bfile SNP-c --logistic --ci 0.95 -- covar covar_age_LUAD_change.txt --covar-number 1 --out SNP-c+adj

Finally, organize the odds ratio (OR) and p-values to identify significant loci.

***Sample size prediction***

The sample size for the case-control study was calculated using the following formula, with a significance level (α) set to 0.05 and a statistical power (1-β) of 0.8. We assumed event rates of 20% for the case group and 10% for the control group, with an effect size (Odds Ratio, OR) of 2.0. p1=0.2 denotes the event rate for the case group, and p2=0.1 is the event rate for the control group. Based on these parameters, the required minimum sample size was determined to be 196 participants per group. The formula used for the calculation is:

$$n=\frac{({Z_{\alpha/2}+Z_{\beta})}^{2}\times(p1\left( 1-p1 \right)+p2\left( 1-p2 \right))}{{(p1-p2)}^{2}}$$

Our case-control study included 3,453 LUAD cases and 3,710 healthy controls, which is much larger than the required sample size of 196. This ensures that the study meets the necessary criteria and provides reliable results for susceptibility analysis.

***DNA extraction and genome-wide DNA methylation detection***

Genomic DNA from inhouse tissue samples was extracted using the QIAamp DNA Mini Kit (Qiagen) and bisulfite-converted with the EZ DNA Methylation-Gold™ Kit (Zymo Research) following the manufacturer’s protocols. Between 500 and 1000 ng of bisulfite-converted DNA was hybridized to the Illumina BeadChip and processed in accordance with the Infinium HD methylation protocol. The resulting Illumina Intensity Data (IDAT) from the 850k BeadChip were further analyzed using the ChAMP package (version 2.26.0) ^1^. The champ.DMP() function operates by applying a linear regression model to the beta values for each CpG site. The beta values, which represent the proportion of methylation at a specific CpG site (ranging from 0 to 1), serve as the dependent variable in the model. The beta values are converted into M-values for statistical testing, as M-values offer improved statistical properties for differential analysis. The following parameters were specified in our analysis:

Comparison groups: "case" vs. "control".

Adjusted p-value threshold: false discovery rate (FDR) < 0.05.

Correction method: Benjamini-Hochberg for multiple testing.

The results of champ.DMP() include adjusted p-values, beta differences, and other statistical metrics for each CpG site, enabling the identification of significantly differentially methylated positions.

***RNA extraction and qRT-PCR***

Total RNA extraction was performed using TRIzol™ LS reagent (Thermo Fisher Scientific), followed by reverse transcription into cDNA with the Prime Script™ RT reagent kit (Takara, #RR047A). Quantitative real-time polymerase chain reactions (qRT-PCR) were conducted using LightCycler^®^ 480 SYBR Green I Master (Roche, UK) on a Roche LightCycler 480 instrument. The thermal cycling conditions were as follows: initial denaturation at 95°C for 10 minutes, followed by 45 cycles of denaturation at 95°C for 10 seconds, annealing at 59°C for 20 seconds, and extension at 72°C for 20 seconds. The final extension included denaturation at 95°C for 10 seconds, annealing at 65°C for 60 seconds, and denaturation at 97°C for 1 second.

***Cell culture***

H1975 cells were maintained in RPMI 1640 medium, while PC9, SPCA-1, and HEK293T cells were cultured in DMEM medium, both supplemented with 10% fetal bovine serum (FBS), 100 μg/mL streptomycin, and 100 U/mL penicillin. All cell lines were incubated at 37°C with 5% CO_2_ after supplementation with the complete medium.

***Generation of overexpression cell lines***

The construction of lentiviral plasmids for this study was carried out by Nanjing Corues Biotechnology Co., Ltd. HEK293T cells were seeded in 10 cm dishes one day prior to packaging to ensure the cells were in an optimal state and density for lentiviral packaging the following day. Following the protocol, the packaging plasmid psPAX2, envelope plasmid PMD2.G, and target gene plasmid PLVX (Lv-NC/ Lv-*LRRC2*) were co-transfected into HEK293T cells using Lipo8000™ transfection reagent (Beyotime, Shanghai, China) at the ratio of 7.5 μg: 2.5 μg: 10 μg, respectively. After 48 hours of incubation, the lentivirus-containing supernatant was collected. Next, the lentivirus supernatant and enhanced transfection reagent Polybrene/polyamine (10 mg/ml) were used to infect the selected H1975 cells, which had been early-planted in the 6cm dishs. After 6 hours, the complete medium containing serum and antibiotics was added. The medium was then replaced after 24 hours, and cells were further incubated for 72 hours before passaging. Simultaneously, puromycin was added to select a stable cell population with resistance. After expanding the selected cells, they were used for subsequent experiments.

***Cell proliferation assay***

A total of 1,000 Lv-NC or Lv-*LRRC2* H1975 cells were seeded into 6-well plates and incubated at 37°C with 5% CO_2_. The RPMI 1640 medium was replaced every 3 days. After one week, cells were fixed and stained with 0.1% crystal violet (Beyotime, Shanghai, China) for 30 minutes, and colony counting was performed using Image J software.

***Transwell migration assay***

In the migration assay, Lv-NC and Lv-*LRRC2* cells were counted and seeded into a serum-free medium in the upper chamber of a Transwell filter (Corning). The lower chamber contained RPMI 1640 supplemented with 10% FBS. After 24 hours, cells were fixed in methanol for 1 hour, stained with crystal violet for 30 minutes, photographed with a fluorescence microscope, and counted using Image J software.

***LRRC2 pathway enrichment analysis and risk analysis in LUAD***

The URLs referenced in this section are as follows: Gene Set Enrichment Analysis (GSEA) (<https://www.gsea-msigdb.org/gsea/index.jsp>); Gene Ontology (GO) (<http://geneontology.org/>); Kyoto Encyclopedia of Genes and Genomes (KEGG) (<https://www.genome.jp/kegg/>); The Database for Annotation, Visualization and Integrated Discovery (DAVID) (<https://david.ncifcrf.gov/>); and PrognoScan database (<http://dna00.bio.kyutech.ac.jp/PrognoScan/>).

We stratified the samples into high and low *LRRC2* expression groups based on median *LRRC2* expression levels, then used this binary classification as the phenotype label. We employed the GSEA software (v4.3.2) developed by the Broad Institute to identify biological pathways and processes that show significant enrichment between these *LRRC2*-high and *LRRC2*-low groups. Furthermore, to perform comprehensive gene set enrichment analysis (GSEA), we strategically selected four curated gene collections from the Molecular Signatures Database (MSigDB) version 2024.1: 1). The Canonical Pathways subset (C2.CP.KEGG_medicus) containing Kyoto Encyclopedia of Genes and Genomes pathways; 2-4). Three Gene Ontology collections encompassing biological processes (C5.GO.BP), cellular components (C5.GO.CC), and molecular functions (C5.GO.MF). This multi-dimensional approach enabled simultaneous interrogation of disease-related pathways (KEGG), functional hierarchies (GO biological processes), subcellular localization patterns (GO cellular components), and molecular activity mechanisms (GO molecular functions), ensuring systematic coverage of biological phenomena at different organizational levels.

As detailed in the main text, a two-sample Mendelian randomization (MR) analysis was employed to explore the causal relationship between *TEK*—a gene highly correlated with *LRRC2*—and LUAD. Instrumental variables (IVs) were selected based on established criteria: relevance assumption, independence assumption, and exclusion restriction assumption. Using the TwoSampleMR package (https://github.com/MRCIEU/TwoSampleMR), seven TEK-associated IVs were identified. The MR-Egger regression method was utilized to assess model robustness and detect potential horizontal pleiotropy. The inverse variance weighted (IVW) method was applied to evaluate the causal association between *TEK* and LUAD.

***Construction of a prognostic model using machine learning on LRRC2-related gene sets***

The study incorporated samples from the TCGA-LUAD cohort (training set) and GSE72094 cohort (validation set), with survival as endpoints. The TCGA-LUAD cohort (N=600) was selected as the training set given its comprehensive multi-omics data, standardized clinical annotations, and established utility in lung adenocarcinoma biomarker discovery. For independent validation, the GSE72094 cohort (N=442) was chosen based on three critical considerations: (1) it represents a distinct patient population profiled on a different sequencing platform (RNA-seq vs. microarray), thereby testing technical generalizability; (2) it provides well-annotated survival outcomes required for prognostic model validation; and (3) it has been widely adopted as an external validation cohort in prior LUAD studies ^2^. To ensure robust internal validation, we implemented dataset-specific harmonization procedures: within the TCGA-LUAD training cohort, batch effects were mitigated using ComBat (Citation) followed by transcripts per million (TPM) normalization, while the GSE72094 validation set underwent internal quality control through RMA-normalized microarray processing prior to downstream analysis. And we performed internal normalization separately for each dataset (**see Figure below**), and ensured gene feature matching across both cohorts-an approach aligned with biomarker research best practices and widely accepted in leading clinical translational journals. A broad range of machine learning algorithms was implemented, including Least Absolute Shrinkage and Selection Operator (LASSO), Gradient Boosting Machine (GBM), Random Survival Forest (RSF), Partial Least Squares Regression for Cox models (plsRcox), Stepwise Cox Proportional Hazards Regression (StepCox), Supervised Principal Components (SuperPC), Ridge Regression, Survival Support Vector Machine (Survival-SVM), Likelihood-Based Boosting for Cox Models (CoxBoost), and Elastic Net (Enet). Algorithms with dimensionality reduction and variable selection functions—such as RSF, LASSO, CoxBoost, and StepCox—were used in the initial steps and combined with other models, resulting in 101 distinct machine learning algorithm combinations.

**
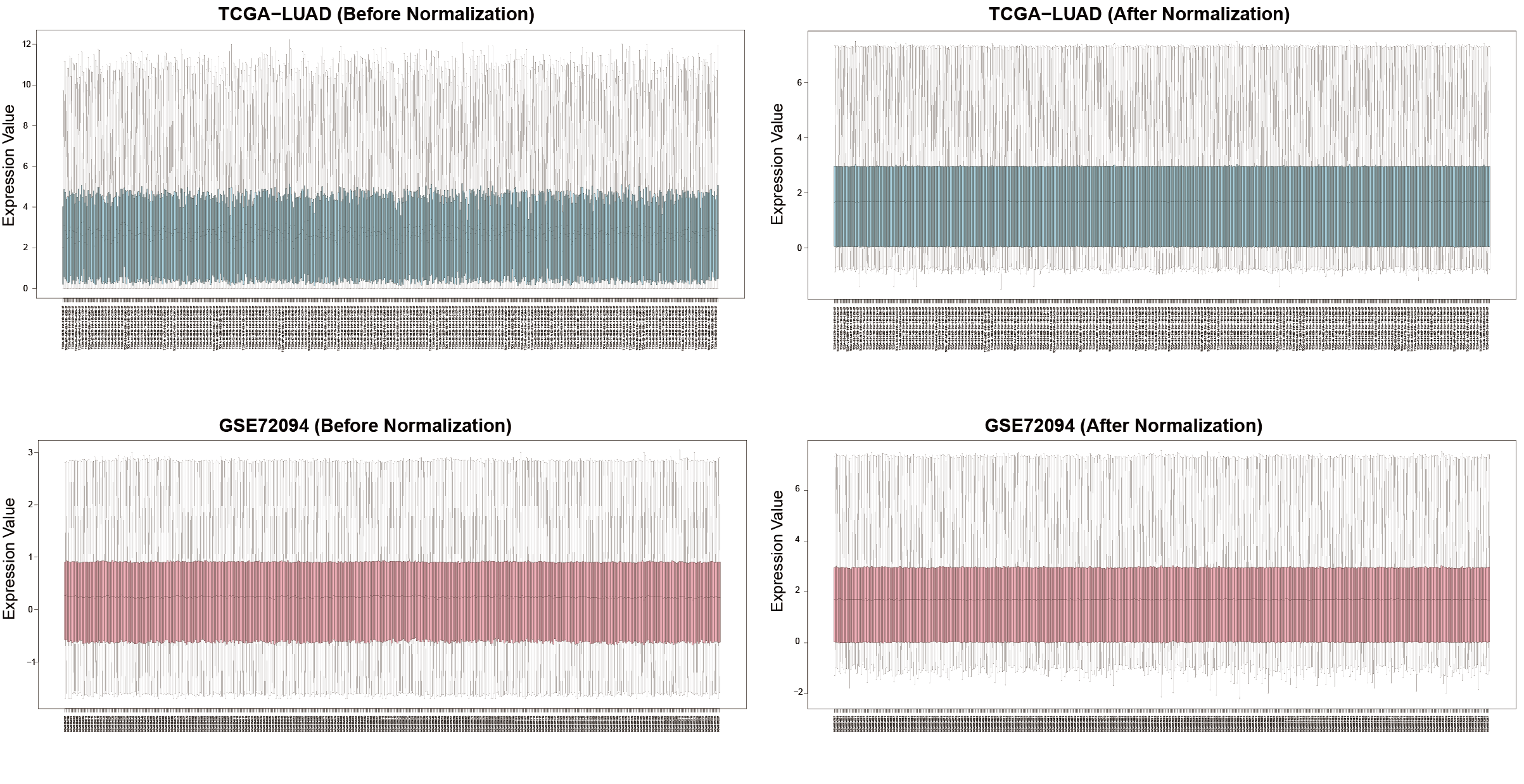
****Normalization of TCGA and GSE72094 cohort**

***Assessment of LRRC2 clinical and radiomics models in LUAD***

Immunohistochemical (IHC) staining was conducted following standard protocols using the *LRRC2* antibody (PA5-58668, ThermoFisher). Target delineation was independently performed by three licensed radiologists, and regions of interest (ROIs) were defined while avoiding areas of necrosis, cystic changes, and calcification. Inter-observer agreement was measured, with a concordance threshold set at > 0.8. Radiomics features were extracted from public database images using PyRadiomics (version 3.0.1; <https://github.com/AIM-Harvard/pyradiomics>).

Significant differences in radiomics features were assessed using Limma analysis (version 3.60.4; <https://bioconductor.org/packages/limma/>).

***Specific meanings of two key poorer survival-related imaging features***

Further analysis of the C1 and C4 groups highlighted two key poorer survival-related imaging features: lbp-3D-k_glrlm_GrayLevelVariance, which reflects variation in gray levels within 3D image textures, gray level variance is a statistical index used to quantify the degree of dispersion of gray value in an image. It reflects the fluctuation of pixel gray value in the image, which can help to analyze the heterogeneity or lesion characteristics of lung tissue. A larger variance indicates a more uneven distribution of gray values in the lung tissue, which may suggest density changes due to lesions such as pulmonary fibrosis, emphysema, or tumors. And lbp-3D-k_glrlm_HighGrayLevelRunEmphasis, which indicates higher gray intensity values in 3D images. In the 3D image of lung tissue, the gray value reflects the density and properties of the tissue. High gray values usually indicate areas of higher density, such as lesions such as fibrosis, calcification, or tumors. A higher concentration of gray values may indicate more significant or extensive lesions in these regions. Both features were associated with mortality risk.

**Supplementary Results**

***The relationships with the surrounding DNA regulatory elements***

To further understand the potential regulatory implications of this epigenetic cluster, we investigated whether these CpG loci-and the associated SNP rs939408-reside within shared genomic regulatory elements. Using the JASPAR database, we identified several transcription factors (TFs) predicted to bind near these loci. Specifically, ZNF354A and Sox6 were predicted to bind across the SNP rs939408, while Nrf1 and Zfp961 were associated with the CpG region near cg09596674. Allele-specific motif analysis showed that the A allele of rs939408 may enhance ZNF354A binding, while the T allele might strengthen Sox6 binding compared to the C allele, suggesting potential allele-specific transcriptional regulation. Similarly, CpG methylation might influence TF accessibility and thereby *LRRC2* gene expression (**Supplementary Figure 7**).

To extend the analysis beyond local elements, we explored the 3D chromatin architecture using publicly available Hi-C and ENCODE datasets. Our results show that both rs939408 and cg09596674 reside within the same Topologically Associating Domain (TAD). TADs are contiguous genomic regions in which loci interact more frequently with each other than with loci outside the domain. TADs are typically 400–800 kb in length and are considered relatively stable structural and functional units of the genome. TAD boundaries are characterized by:

(1) Sharp drops in interaction frequencies, defined by Directionality Index (DI) values approaching zero;

(2) High enrichment of architectural proteins such as CTCF, and active chromatin marks like H3K4me3 and H3K36me3;

(3) Epigenetic signatures associated with housekeeping genes, tRNA genes, and SINEs.

TADs regulate gene expression primarily by constraining enhancer-promoter interactions within their own boundaries, creating insulated regulatory environments. Current models suggest that TAD formation involves loop extrusion mediated by cohesin complexes and anchored by CTCF binding sites. These structural features maintain transcriptional fidelity and limit inappropriate regulatory cross-talk. Subdomains within TADs (sub-TADs), which are ~100 kb in length, may exhibit cell-type specificity and further refine enhancer-promoter communication.

The co-localization of rs939408 and cg09596674 within the same TAD strongly supports the hypothesis that:

(1) These elements are part of a shared regulatory landscape;

(2) Their spatial proximity may enable coordinated modulation of *LRRC2* expression;

(3) Methylation or allelic variation at these loci may alter chromatin conformation or affect the binding dynamics of TFs, ultimately regulating the expression of *LRRC2*.

***Interaction Analysis analysis on the potential interactions between different meQTLs, genes, and environmental factors***

Given that meQTLs involve both SNPs and CpG sites, we considered their biological nature when designing our interaction analyses. SNPs represent germline variations that are generally stable and unlikely to be influenced by environmental factors. In contrast, CpG methylation levels are more dynamic and susceptible to environmental factors such as smoking. Therefore, in the subsequent analyses, we focused on potential interactions between CpG methylation, gene expression, and environmental factors (smoking).

Specifically, as shown in the **Supplementary Figure 9**, we first stratified the TCGA samples by smoking status to explore whether smoking modified the relationship between CpG methylation and gene expression **(Supplementary Figure 9A)**. The scatterplots revealed that the trends of correlation between cg09596674 methylation level and *LRRC2* gene expression level were consistent across smokers and non-smokers, without distinct separation or aggregation into independent regions. This observation suggested a lack of substantial interaction between smoking exposure and the cg09596674 methylation-*LRRC2* gene expression relationship.

To further verify this, we conducted subgroup analyses stratified by gender, including the total samples (combined males and females), males only, and females only. Consistently, no significant differences were observed in cg09596674 methylation levels between smokers and non-smokers in the overall population **(Supplementary Figure 9B up)** or among males **(Supplementary Figure 9D up)**, while a slight difference was noted among females **(Supplementary Figure 9F up)**. Besides, no significant differences were observed in *LRRC2* gene expression levels between smokers and non-smokers in the overall population **(Supplementary Figure 9B down)**, among males **(Supplementary Figure 9D down)**, or among females **(Supplementary Figure 9F down)**.

Importantly, across all stratifications by overall samples **(Supplementary Figure 9C)**, males only **(Supplementary Figure 9E)**, and females only **(Supplementary Figure 9G)**, the negative correlation between cg09596674 methylation level and *LRRC2* gene expression level remained statistically significant and consistent, regardless of smoking status. These results indicate that environmental exposure (smoking) may not materially modify the negative regulatory relationship between cg09596674 methylation and *LRRC2* gene expression, and thus may not play a major interactive role in LUAD development in this context.

Additionally, as presented in **Supplementary Figure 10**, we conducted a further stratified analysis by categorizing participants into high and low methylation groups based on the median methylation level of the cg09596674 site and then cross-classified them by smoking status. Consistently, we found that significant differences in *LRRC2* gene expression levels only appeared when comparing high versus low cg09596674 methylation groups within the same smoking status (either smokers or non-smokers), but not between smoking and non-smoking groups. This further corroborates the absence of an interaction between environmental factors (smoking) and the cg09596674 methylation effect on *LRRC2* gene expression.

Finally, our formal interaction model analysis (forest plot) also confirmed the lack of significant interaction effects between cg09596674 methylation, *LRRC2* gene expression, and smoking, providing additional validation of our findings **(Supplementary Figure 11)**.

# Reference

1. Wang Z, Zhao Y, Phipps-Green A, Liu-Bryan R, Ceponis A, Boyle DL*, et al.* Differential DNA Methylation of Networked Signaling, Transcriptional, Innate and Adaptive Immunity, and Osteoclastogenesis Genes and Pathways in Gout. *Arthritis & rheumatology (Hoboken, NJ)* 2020, **72**(5)**:** 802-814.

2. Chang W, Li H, Zhong L, Zhu T, Chang Z, Ou W*, et al.* Development of a copper metabolism-related gene signature in lung adenocarcinoma. *Frontiers in immunology* 2022, **13:** 1040668.

**
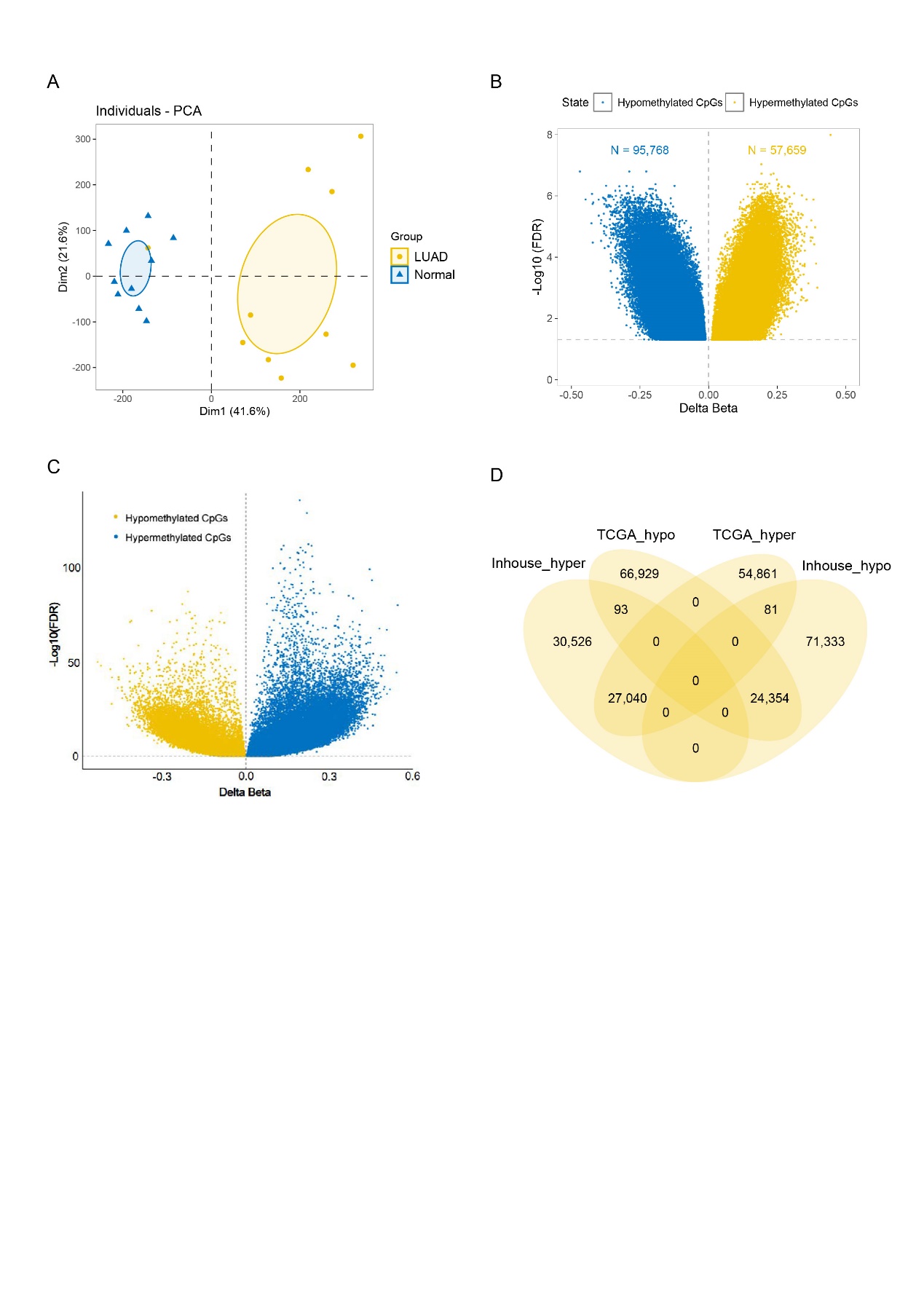
**

# Supplementary Figure 1 Quality control and differential methylation CpG site analysis

(A) Principal component analysis of inhouse methylation array data.
(B) Volcano plot displaying the differentially methylated CpG sites identified in the inhouse methylation array data.
(C) Volcano plot showing the differentially methylated CpG sites in the TCGA methylation array data.
(D) Venn diagram illustrating the overlap of methylated CpG sites between the inhouse LUAD dataset and the TCGA LUAD dataset.


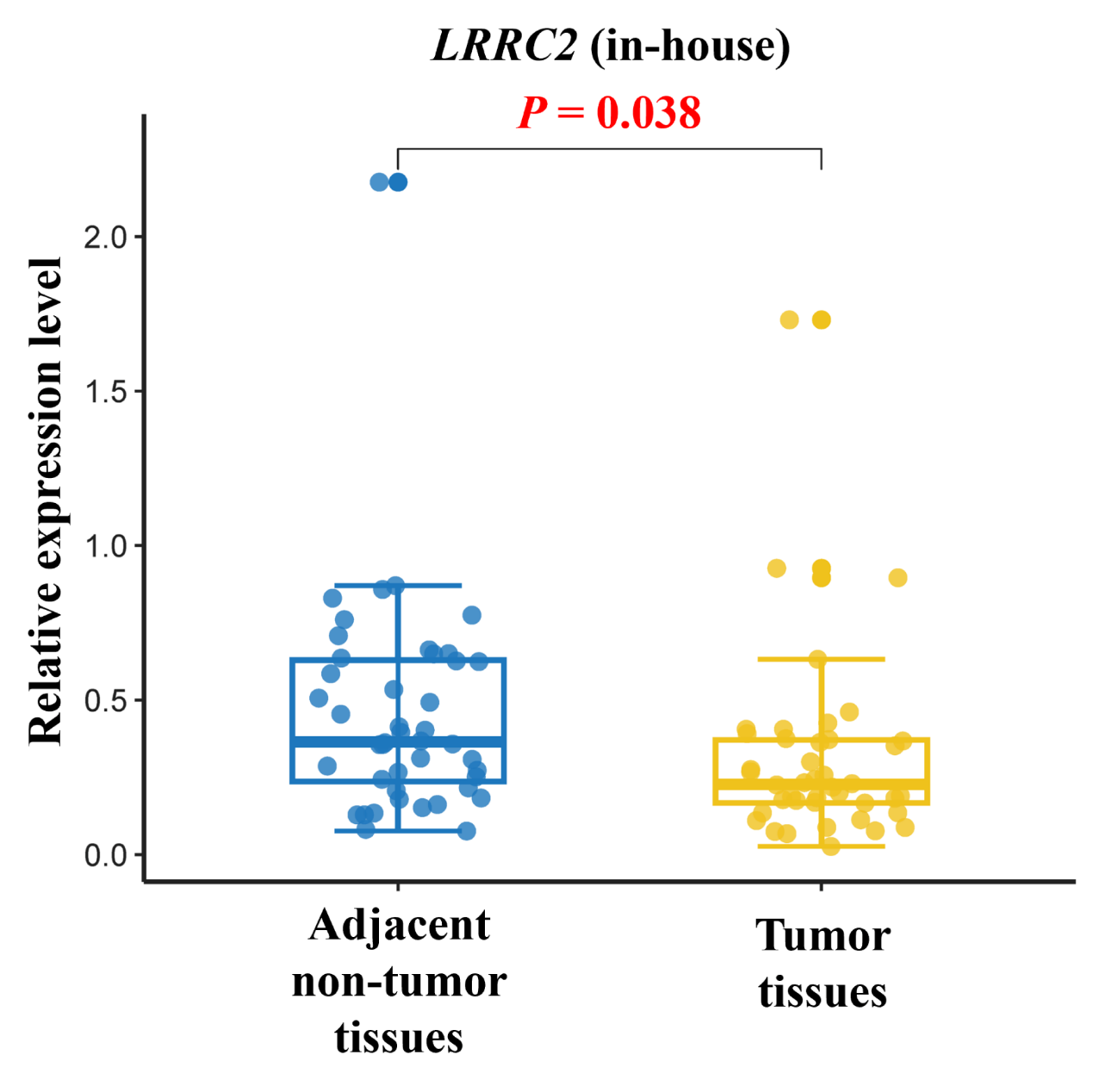


**Supplementary Figure 2 Differential expression levels of *LRRC2* in expanded 41 paired LUAD tissue samples**

**
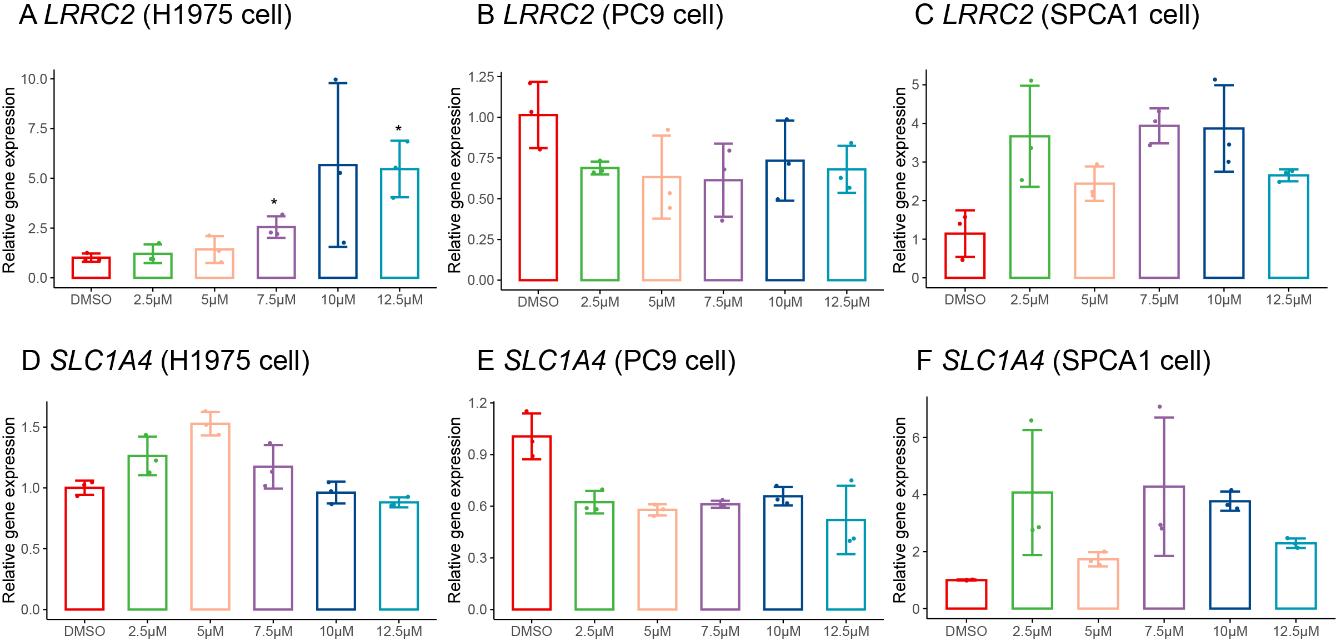
**

# Supplementary Figure 3 Comparison of gene expression between control and 5-Aza treated groups.

The relative mRNA expression of target genes was measured after treatment with a range of 5-Aza concentrations in H1975, SPCA1, and PC9 cells.


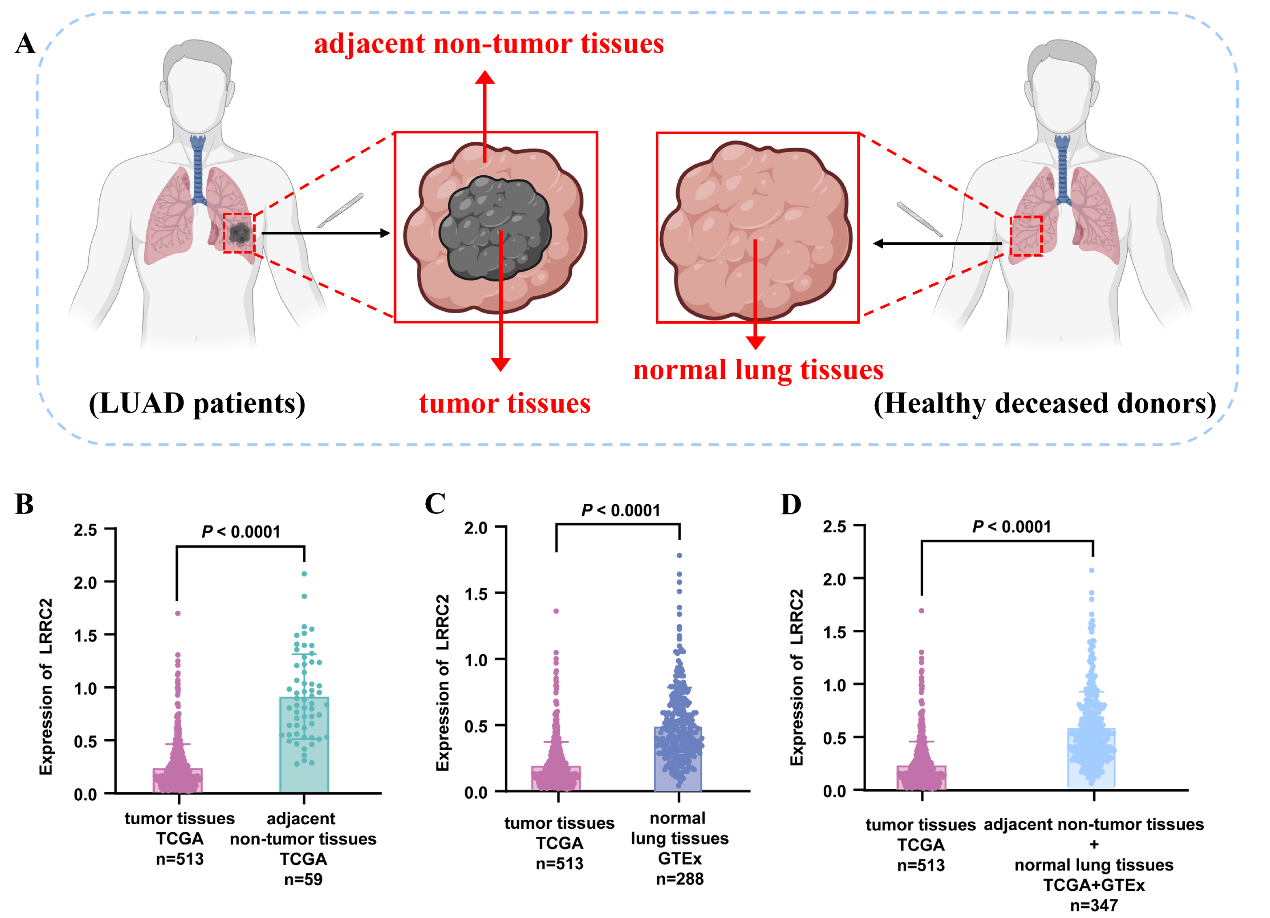


**Supplementary Figure 4 In-depth analysis of differential *LRRC2* expression between tumor and normal lung tissues**(A) Schematic overview of LUAD tumor tissues, adjacent non-tumor tissues, and normal lung tissues sampling.
(B) Differential expression of *LRRC2* between LUAD tumor tissues and adjacent non-tumor tissues.
(C) Differential expression of *LRRC2* between LUAD tumor tissues and normal lung tissues.
(D) Differential expression of *LRRC2* between LUAD tumor tissues and the combined group of adjacent non-tumor tissues and normal lung tissues.
Abbreviation: LUAD, lung adenocarcinoma.


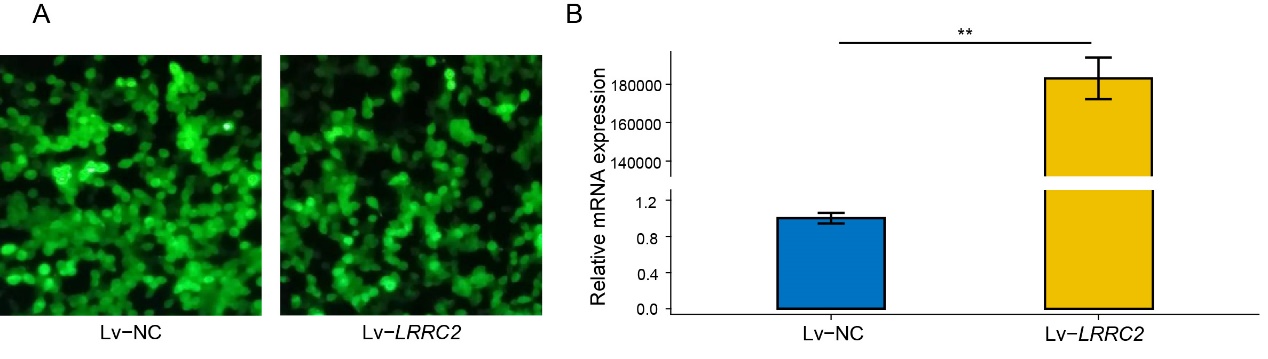


# Supplementary Figure 5 Successful generation of stable cell lines.

(A) Fluorescence microscopy images showing the successful generation of control Lv-NC and overexpression Lv-*LRRC2* stable cell lines.
(B) Quantitative analysis of *LRRC2* mRNA expression in Lv-NC and Lv-*LRRC2* cell lines.


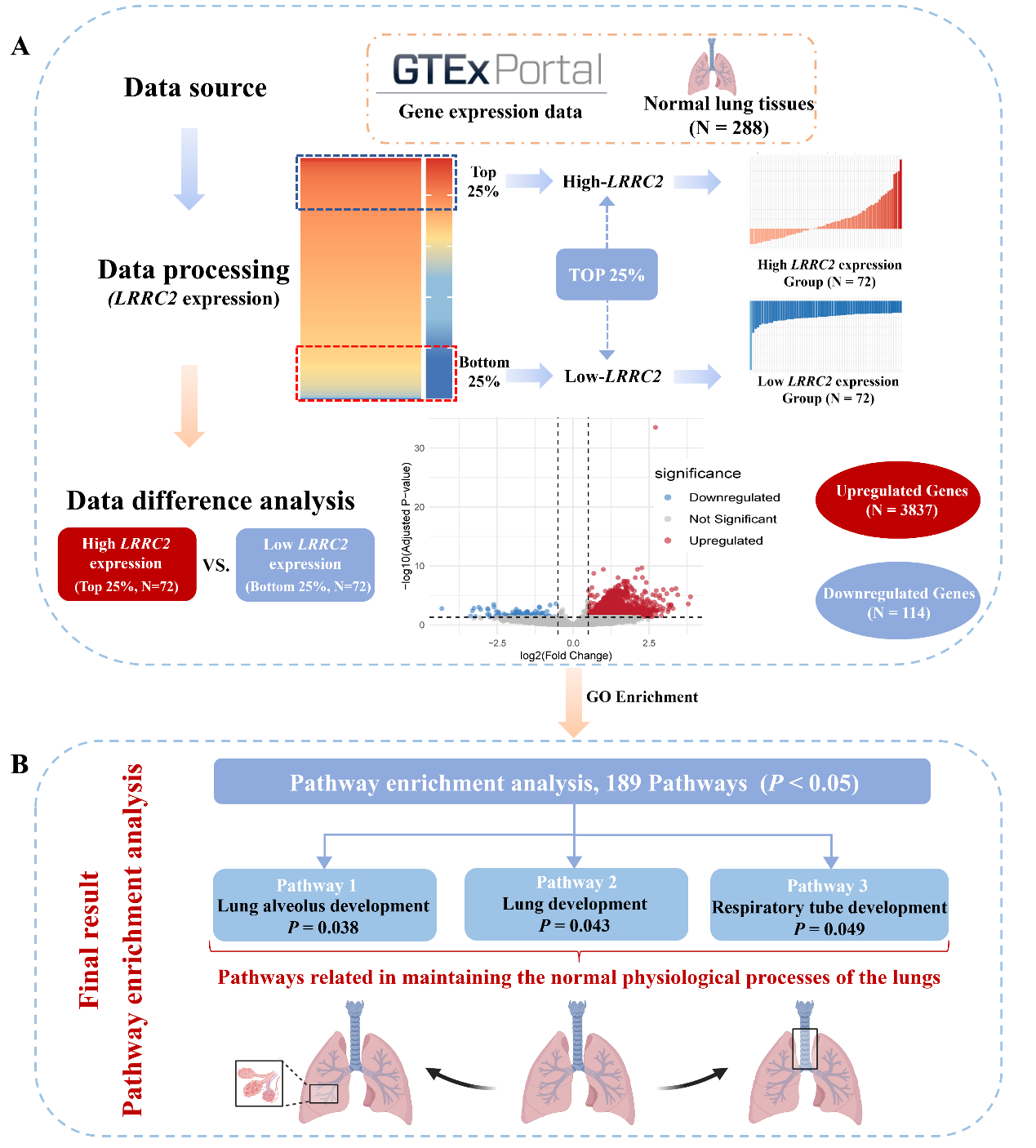


**Supplementary Figure 6 Difference expression analysis and functional annotation of *LRRC2* in normal lung tissue.**

(A) To explore the biological role of *LRRC2* in normal lung physiology, GTEx normal lung tissue samples (N = 288) were stratified into high (top 25%) and low (bottom 25%) *LRRC2* expression groups. Differential expression analysis identified 3,837 upregulated and 114 downregulated genes, laying the foundation for subsequent functional enrichment analysis.

(B) Enrichment analysis based on the differentially expressed genes identified a total of 189 significantly enriched pathways (*P* < 0.05), encompassing lung-specific physiological processes such as alveolus development, lung development, and respiratory tube development, suggesting that *LRRC2* may play an important regulatory role in maintaining normal lung function.


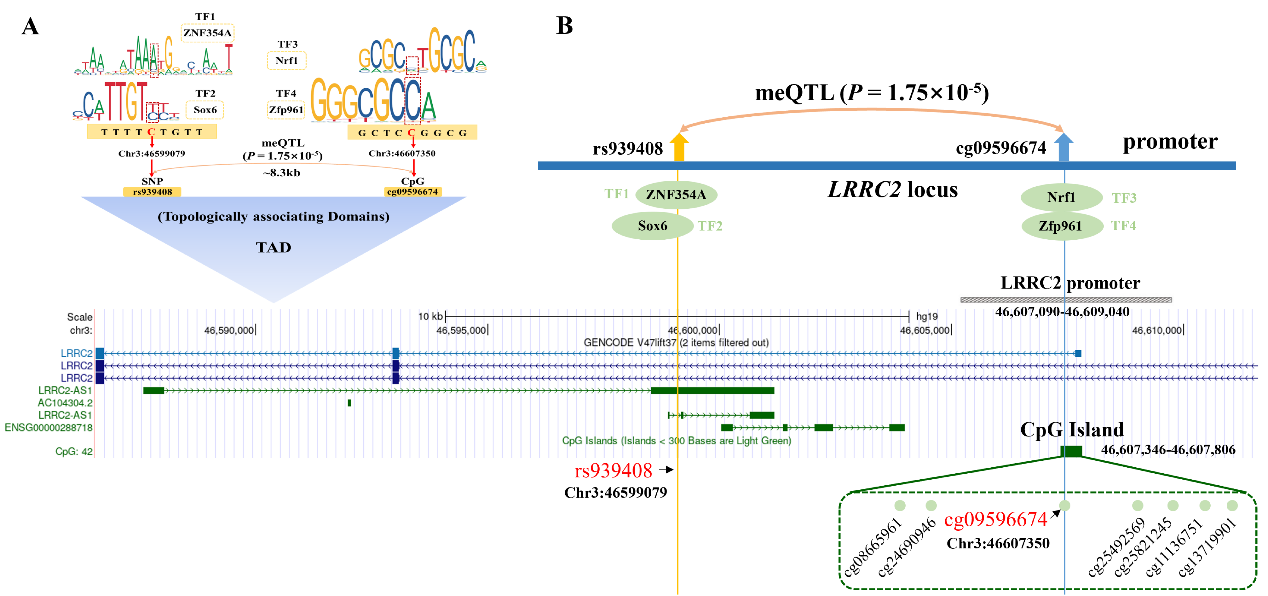


**Supplementary Figure 7 Functional annotation and transcription factor binding analysis of rs939408 and cg09596674 within the *LRRC2* regulatory region.**

(A) Transcription factor binding prediction and TAD localization of rs939408 and cg09596674.

(B) Integrative genomic visualization of rs939408 and cg09596674 within the *LRRC2* regulatory landscape.

Abbreviation: TAD, topologically associating domain.


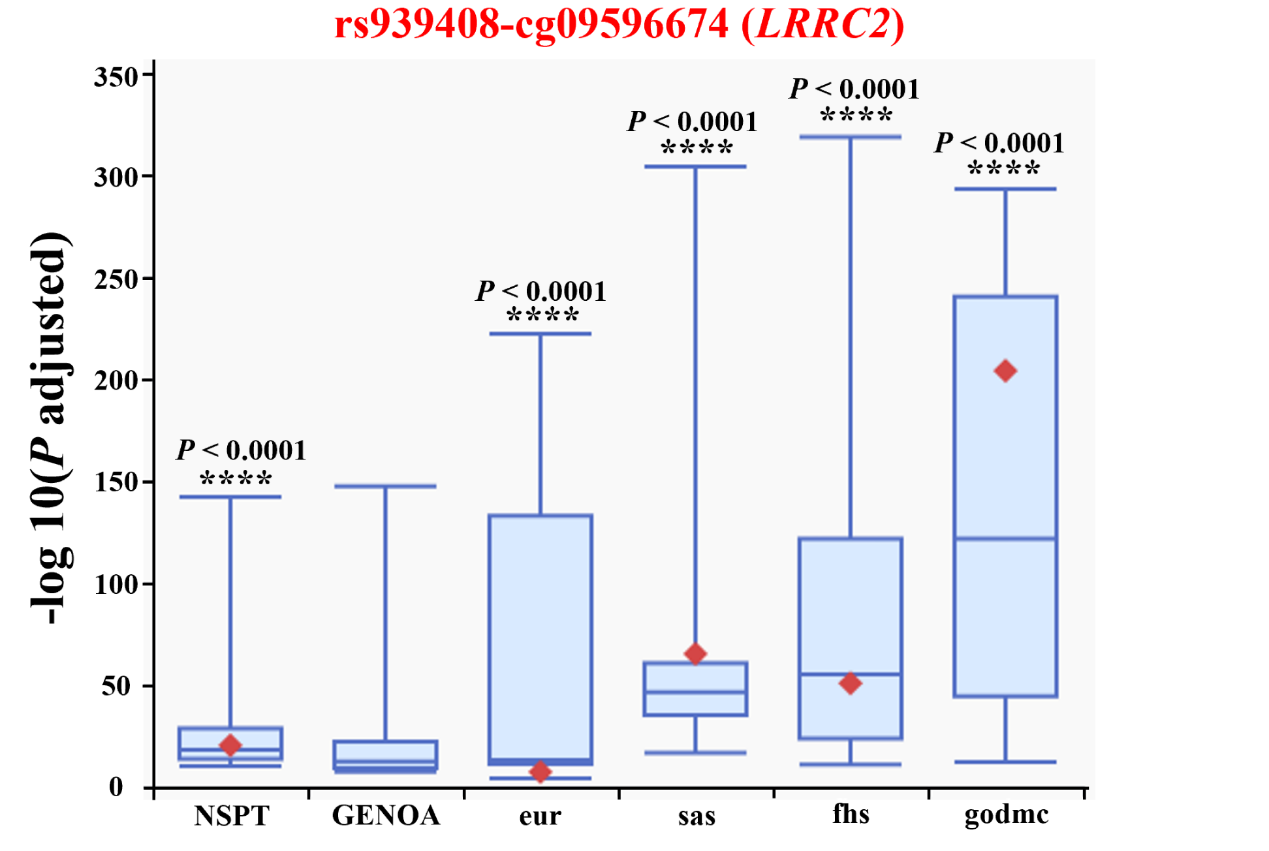


**Supplementary Figure 8 Validation of the positive meQTL in six independent and ethnically diverse meQTL datasets from Sino-mQTL. (**[**https://www.biosino.org/sinomqtl/browse**](https://www.biosino.org/sinomqtl/browse)**).**

NSPT (including 3,523 Chinese from the National Survey of Physical Traits cohort);

GENOA (Genetic Epidemiology Network of Arteriopathy, a multi-ethnic cohort from the U.S.);

eur (European ancestry from the Pan-meQTL database);

sas (South Asian ancestry from the Pan-meQTL database);

fhs (Framingham Heart Study, a long-standing European ancestry cohort);

godmc (an international collaboration of human epidemiological studies that comprises >30,000 study participants with genetic and DNAm data).


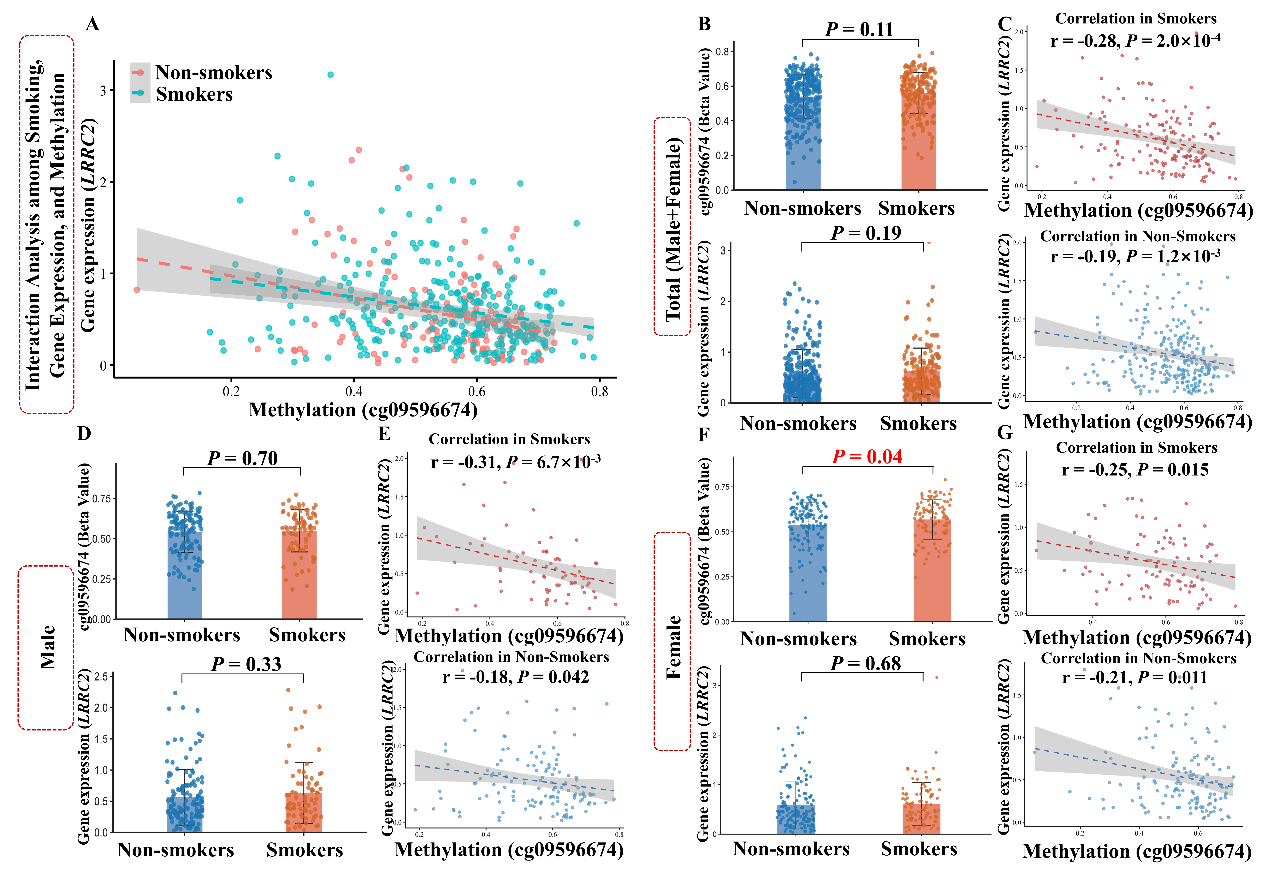


**Supplementary Figure 9 Interaction analysis between smoking status, cg09596674 methylation, and *LRRC2* gene expression**

(A) Scatterplots illustrating the correlation between cg09596674 methylation and *LRRC2* expression levels among smokers and non-smokers in the overall TCGA LUAD dataset.

(B) Comparison of cg09596674 methylation levels (top) and *LRRC2* gene expression levels (bottom) between smokers and non-smokers in the overall TCGA LUAD dataset.

(C) Correlation between cg09596674 methylation and *LRRC2* expression levels between smokers and non-smokers in the overall TCGA LUAD dataset.

(D) Comparison of cg09596674 methylation levels (top) and *LRRC2* expression levels (bottom) between smokers and non-smokers among males.

(E) Correlation between cg09596674 methylation and *LRRC2* expression levels between smokers and non-smokers among males.

(F) Comparison of cg09596674 methylation levels (top) and *LRRC2* expression levels (bottom) between smokers and non-smokers among females.

(G) Correlation between cg09596674 methylation and *LRRC2* expression levels between smokers and non-smokers among females.


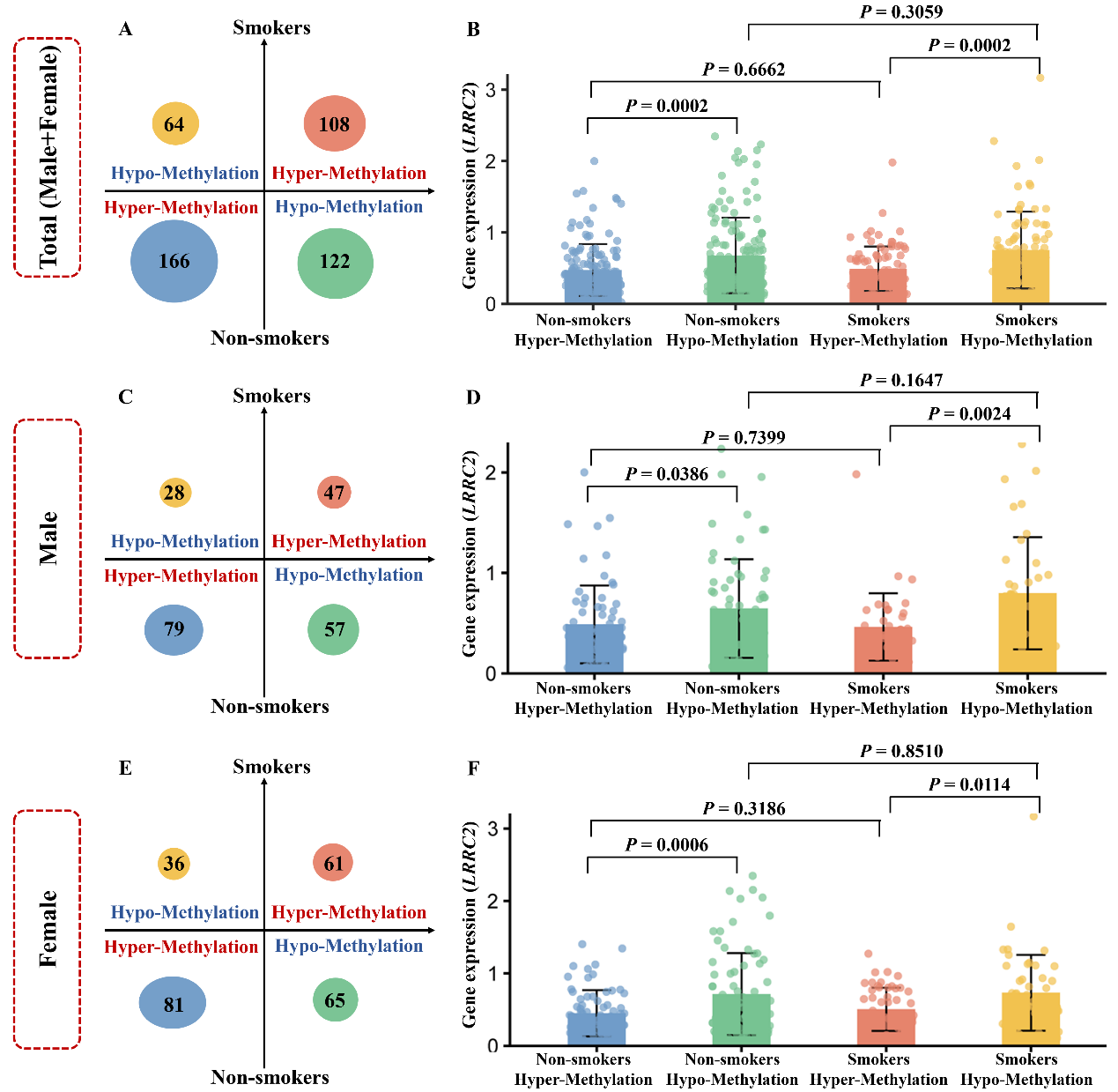


**Supplementary Figure 10 Joint stratified analysis based on CpG methylation levels and smoking status**

(A-B) Comparison of *LRRC2* gene expression levels across four stratified groups based on cg09596674 methylation status (high vs. low) and smoking status (smoker vs. non-smoker) among all participants.

(C-D) Comparison of *LRRC2* gene expression levels across four stratified groups based on cg09596674 methylation status (high vs. low) and smoking status (smoker vs. non-smoker) among males.

(E-F) Comparison of *LRRC2* gene expression levels across four stratified groups based on cg09596674 methylation status (high vs. low) and smoking status (smoker vs. non-smoker) among females.


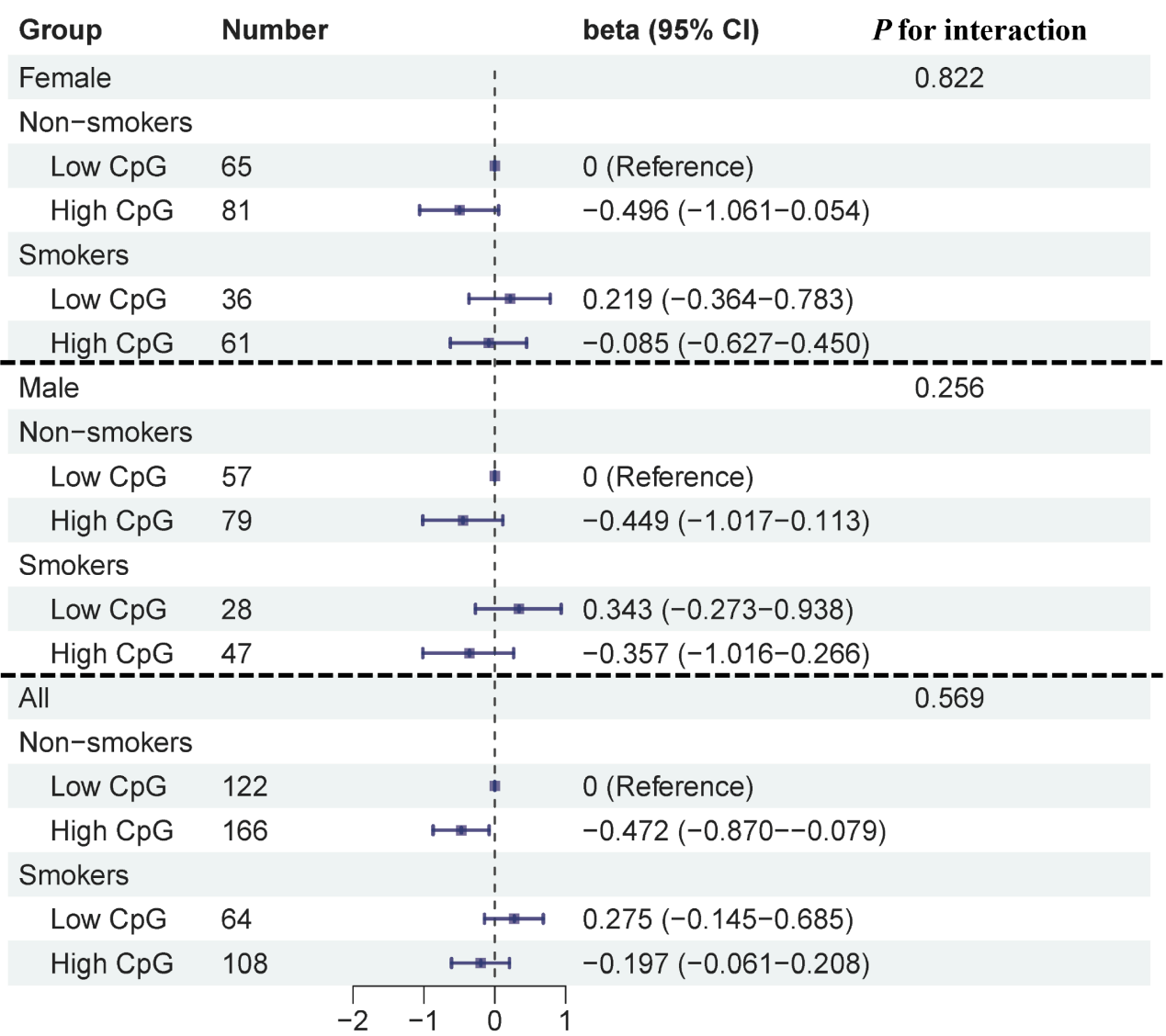


**Supplementary Figure 11 Comprehensive interaction analysis of environmental factors, cg09596674 methylation levels, and *LRRC2* gene expression**

To evaluate whether environmental exposure (smoking) modifies the regulatory relationship between cg09596674 methylation and *LRRC2* gene expression, we performed stratified analyses across three subgroups: the overall population, males only, and females only. Participants were categorized into four groups based on smoking status (smoker vs. non-smoker) and cg09596674 methylation level (high vs. low, defined by median split). Forest plots display the estimated effect sizes (β coefficients with 95% confidence intervals) of cg09596674 methylation on *LRRC2* expression within each subgroup.

# Supplementary Table 1: Basic clinical information of samples in this study

| Variables | TCGA-LUAD | GSE31210 | In-house LUAD | Local LUAD samples | LUAD GWAS | |
| --- | --- | --- | --- | --- | --- | --- |
|  | Case  (n = 519) | Case  (n = 226) | Case  (n = 10) | Case  (n = 69) | LUAD  (n = 3453) | Control  (n =3710) |
| Age, n (100%)  ≤60  >60  Unknown |  |  |  |  |  | |
|  | 160 (30.83%)  340 (65.51%) | 108 (47.79%)  118 (52.21%) | **-**  10 (100%) | 24 (34.78%)  45 (65.22%) | 1669 (48.33%)  1784 (51.67%) | 2013 (54.26%)  1697 (45.74%) |
|  | 19 (3.66%) | - | **-** | - | - | - |
| Gender  Female  Male | 279 (53.76%)  240 (46.24%) | 121 (53.54%)  105 (46.46%) | 7 (70%)  3 (30%) | 50 (72.46%)  19 (27.54%) | -  **-** | -  **-** |
| Stage, n (100%)  I  II  Ⅲ/Ⅳ  Unknown | 278 (53.56%)  123 (23.70%)  111 (21.39%)  7 (1.35%) | 168 (74.34%)  58 (25.66%)  **-**  **-** | 10 (100%)  -  -  - | 53 (76.81%)  5 (7.25%)  11 (15.94%)  - |  | |
| Pathologic T, n (100%)  T1/T2  T3/T4  Unknown | 452 (87.09%)  64 (12.33%)  3 (0.58%) |  |  | 68 (98.55%)  1 (1.45%)  - |  | |
| Pathologic N, n (100%)  N0  N1  N2/N3  Unknown | 332 (63.97%)  98 (18.89%)  77 (14.83%)  12 (2.31%) |  |  | 60 (86.96%)  5 (7.25%)  4 (5.80%)  - |  | |
| Pathologic M, n (100%)  M0  M1  Unknown | 352 (67.82%)  25 (4.82%)  142 (27.36%) |  |  | 62 (89.86%)  7 (10.14%)  - |  | |

# Supplementary Table 2: Primers used in qRT-PCR experiments

| Gene | Primer Sequence (Forward) | Primer Sequence (Reverse) |
| --- | --- | --- |
| *LRRC2* | 5’-CAAGTTGACCTACCTTCCCTATTCC-3’ | 5’-GCCATCTTCACATTGGGCATTA-3’ |
| *SLC1A4* | 5’-CATGGACGGAGCAGCCATC-3’ | 5’-GGCAGTCACTAGAATGGTGAAAA-3’ |
| β-actin | 5’-CATGTACGTTGCTATCCAGGC -3’ | 5’-CTCCTTAATGTCACGCACGAT-3’ |

# Supplementary Table 3: Details of CpG Sites corresponding to the 7 selected meQTLs

| No | SNP | CpG | Gene | CpG Location | Strand | Relation to Island | UCSC refGene group |
| --- | --- | --- | --- | --- | --- | --- | --- |
| 1 | rs66719815 | cg19220282 | *SLC1A4* | Chr2:65217528 | + | shore | Body |
| 2 | rs939408 | cg09596674 | *LRRC2* | Chr3:46607350 | - | island | 5'UTR |
| 3 | rs12680375 | cg10700718 | *MYOM2* | Chr8:1992719 | + | opensea | TSS1500 |
| 4 | rs328890 | cg04065210 | *DPY19L1* | Chr7:35074628 | - | shore | Body |
| 5 | rs750373 | cg04571833 | *RAPGEF4* | Chr2:173836756 | + | opensea | Body |
| 6 | rs3743281 | cg16110827 | *SEMA6D* | Chr15: 48056943 | - | opensea | Body |
| 7 | rs2885221 | cg03230154 | *ZNF492* | Chr19: 22817176 | - | shore | 1stExon |
